# Recent Advances in the Molecular Mechanisms of Bacterial Anti‐Phage Defence Systems

**DOI:** 10.1111/1751-7915.70416

**Published:** 2026-08-03

**Authors:** Harald Brüssow

**Affiliations:** ^1^ Department of Neuro‐Urology Balgrist University Hospital, University of Zurich Zurich Switzerland; ^2^ Spinal Cord Injury Center Balgrist University Hospital, University of Zurich Zurich Switzerland

## Abstract

Deep learning models based on both distant protein sequence homology and genetic neighbourhood context searches predicted that 1.5% of bacterial genes or 30 genes per *E. coli* genome represent anti‐phage systems (APS); many are colocalized on defence islands or are found on mobile DNA elements. Thousands of APS thus remain to be defined molecularly. Selected recent examples highlighting an astonishing molecular diversity of these defence systems are described in this editorial. The mechanisms include allosterically regulated dGTPase responding to competing nucleotide signals (Clover); bacterial defence systems activated by phage anti‐defence manoeuvres (Panoptes); a prophage encoded tRNA nuclease activated by a phage tail tip protein (HepS); a helicase‐nuclease complex that scans for ssDNA 3′ overhangs created by phage DNA transaction (Hachiman); and systems that cleave free ends of linear DNA (Shedu). Systems were described that synthesize template‐free poly‐dA chains which are degraded by a phage exonuclease thereby activating an ion channel (Hailong). Several systems interfere with phage DNA injection into the cell, for example, a cell membrane associated protein complex inhibiting injection (KIWA); or destroying phage DNA at injection (SNIPE); or a complex membrane motor system that senses phage DNA injection and activates nuclease effectors (Zorya). Other systems consist of a single pore building protein that combines sensor and effector functions (Rip1) or degrade NAD^+^ (Cat1). Defence‐associated reverse transcriptase (DRT) preceded by non‐coding RNA (ncRNA) come in different forms: in DRT2 a rolling circle reverse transcription leads to an endless protein inducing cell dormancy; in DRT3 a mixed templated and untemplated repeat DNA synthesis becomes cytotoxic in presence of a phage protein; DRT9 synthesizes a polyA strand that might sequester a phage protein needed for DNA replication. Striking are bacterial APS that resemble innate immune reaction directed against viruses in animals (gasdermin, Argonaute, RAZR, Schlafen, Thoeris 2, ubiquitin‐like proteins) pointing to an ancient origin.

## Graphical abstract

Panoptes as an example for an antiphage system consists of OptS producing a cyclic dinucleotide that prevents activation of OptE, a transmembrane protein. Phage T4 infection introduces the sponge protein Acb2 that adsorbs the cyclic dinucleotide thus activating OptE which disrupts the cell membrane aborting phage infection. Figure credit: Sullivan et al. (2025) under a Creative Commons Attribution License 4.0.

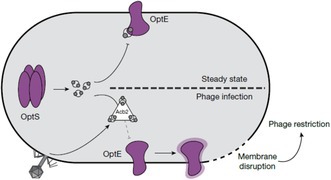

In the mid‐1990s, only a handful of academic laboratories worked with phages. The situation changed dramatically when dairy microbiologists discovered that Clustered Regularly Interspaced Short Palindromic Repeats (CRISPR) provide adaptive immunity against phages in streptococci (Barrangou et al. 2007). Subsequent exploration of the CRISPR‐Cas systems offered biotechnological tools that can only be compared to the introduction of restriction enzymes (also from phage research) and led to a Nobel prize award to J. Doudna and E. Charpentier in 2020. Molecular characterization of bacterial antiphage systems (APS) and the countermeasures by phages led to a renaissance of phage research (Ofir and Sorek 2018). There are a number of excellent reviews by lead scientists on APS (just to quote Annual Reviews: Lopatina et al. 2020; Athukoralage and White 2022; Slavik and Kranzusch 2023; Getz and Maxwell 2024; Hobbs and Kranzusch 2024). The field is developing very rapidly justifying a review of selected reports published over the last 24 months highlighting the astonishing diversity of molecular mechanisms underlying bacterial APS.

## 1 Bioinformatic Predictions of APS

Comprehensiveness is hard to achieve since about 250 APS have been experimentally validated. Recent bioinformatic approaches suggested that the true number of these systems is even much higher. French researchers developed three deep learning methods to predict anti‐phage functions (Mordret et al. 2026). In a genomic context‐based model, defence systems tend to colocalize in bacterial genomes on defence islands, mostly as MGE (mobile genetic element: prophages, satellites, integrons). In protein language models, predictions relied primarily on remote sequence homology to proteins with demonstrated anti‐phage function. Selected predictions were experimentally verified in *Streptomyces* and *Escherichia coli* . The two models are complementary: the context model uncovers systems with no detectable sequence homology to known defence systems while the protein language model reveals homologies across the entire proteome. The model learned that gene's identity can be inferred both from its sequence and from the genomic neighbourhood. The combined model predicted 2 million mostly single antiphage proteins and 20,000 anti‐phage operon families when screening 32,000 bacterial genomes. Most of the identified defence systems were undefined: 400,000 of the protein families lacked any annotation. About 1.5% of genes in bacterial genomes are dedicated to antiphage defence; an association was detected between MGE and phage defence.

Also the DefensePredictor developed by US researchers uses as input the sequence of a gene and its genomic neighbourhood. When screening the genomes of 69 *E. coli* strains, the program predicted 1991 candidate defence genes. Experimental validation of 94 predicted transcription units against a panel of 24 coliphages showed that half of them indeed displayed phage inhibition. When extending the analysis to the pangenome of 3000 *E. coli* strains, the program found 32 defence genes per genome without reaching saturation; most defence genes resided in the accessory pangenome. A similar number of defence proteins per genome was detected when screening 1000 randomly selected bacterial genomes. The researchers studied 45 predicted defence systems consisting of one, two or three genes. The program predicted domains and catalytic sites; mutational analysis demonstrated that they were indeed essential for phage defence. Essential phosphodiesterases, phosphatases, phospholipases and novel toxin‐antitoxin systems were identified (DeWeirdt et al. 2026). The researchers concluded that thousands of APS remain to be defined only in *E. coli* likely to reveal a multitude of different anti‐phage molecular mechanisms. In the following a selection of newly defined anti‐phage mechanisms will be described.

## 2 Clover: Allosterically Regulated dGTPase Responding to Competing Nucleotide Signals

**FIGURE 1:**
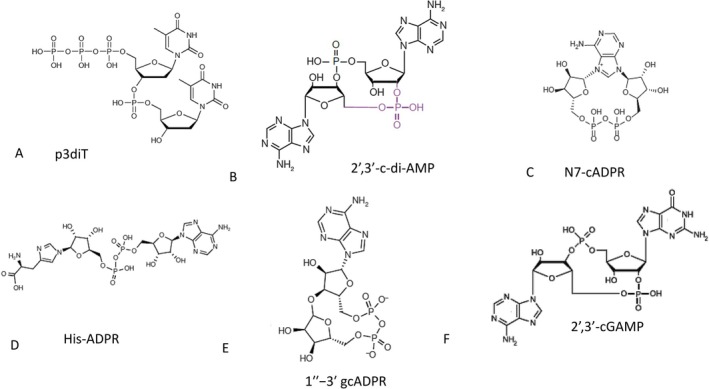
Chemical structure of nucleotide signalling molecules involved in the anti‐phage systems discussed in the present editorial review. (A) The thymidine di‐nucleotide in 3′–5′ phosphodiester linkage (p3diT) used in the Clover APS described by Yamaguchi et al. (2026). (B) The cyclic dinucleotide 2′,3′‐c‐di‐AMP produced by OptS inhibiting OptE in the Panoptes APS described by Sullivan et al. (2025). (C) N7‐cADPR signal used in the Thoeris APS described by Rousset et al. (2025). (D) The His‐ADPR signal used in the Thoeris APS described by Sabonis et al. (2025). (E) The glycocyclic gcADPR signal used in the Thoeris APS described by Tamulaitiene et al. (2024). (F) The CBASS signal 2′,3′‐cGAMP described by Gao et al. (2026).

When bioinformatically analysing bacterial anti‐phage defence islands, a two gene constellation consisting of a deoxynucleoside triphosphohydrolase enzyme (dNTPase) and nucleotidyltransferase (NTase) was detected. Researchers cloned this system from *Salmonella* and *E. coli* which conferred a strong resistance to coliphage T5 (Yamaguchi et al. 2026). A mutation introduced into the active site of the dNTPase (CloA) abolished phage resistance, while a mutated NTase (CloB) killed the cell. How does this system work? *E. coli* when infected with phage T5 increases intracellular dNTP concentrations to satisfy the phage DNA replication needs. In vitro experiments demonstrated that CloA was allosterically activated by the elevated dTTP concentration induced by phage infection. Activated CloA becomes a dGTPase which hydolyzes the dGTP precursor needed for phage replication, but would also kill the cell by decreasing the physiological dGTP pool. The researchers hypothesized that Clo B NTase might restrain CloA activity by synthesizing a signalling molecule that binds and inhibits CloA. By co‐expressing CloA and CloB and analysing the CloA structure (if forms an octamer resembling a four‐leaf clover, hence the name of this APS), they observed an additional nucleotide ligand bound to CloA. Elution of this ligand, followed by chemical analysis identified a thymidine di‐nucleotide in 3′‐5′ phosphodiester linkage (p3diT) (Figure 1A). Mutating the catalytic site of CloB abolished the synthesis of this p3diT ligand. The researchers then compared the structure of the activated CloA–dGTP–dTTP with the suppressed CloA–dGTP–p3diT protein. They identified three separate nucleotide binding sites: dGTP in the active site for enzymatic hydrolysis and dTTP and p3diT bound to two separate allosteric sites, activating or inhibiting the enzyme, respectively. The CloA‐CloB system thus represents a constitutive negative regulation to restrain effector proteins in the absence of phage infection. Manipulation of the nucleotide pool by the infecting phage actually triggers the system. A 20‐fold higher dTTP concentration over p3diT is needed to activate CloA.

## 3 Panoptes: Bacterial Defence Detecting Phage Anti‐Defence

**FIGURE 2:**
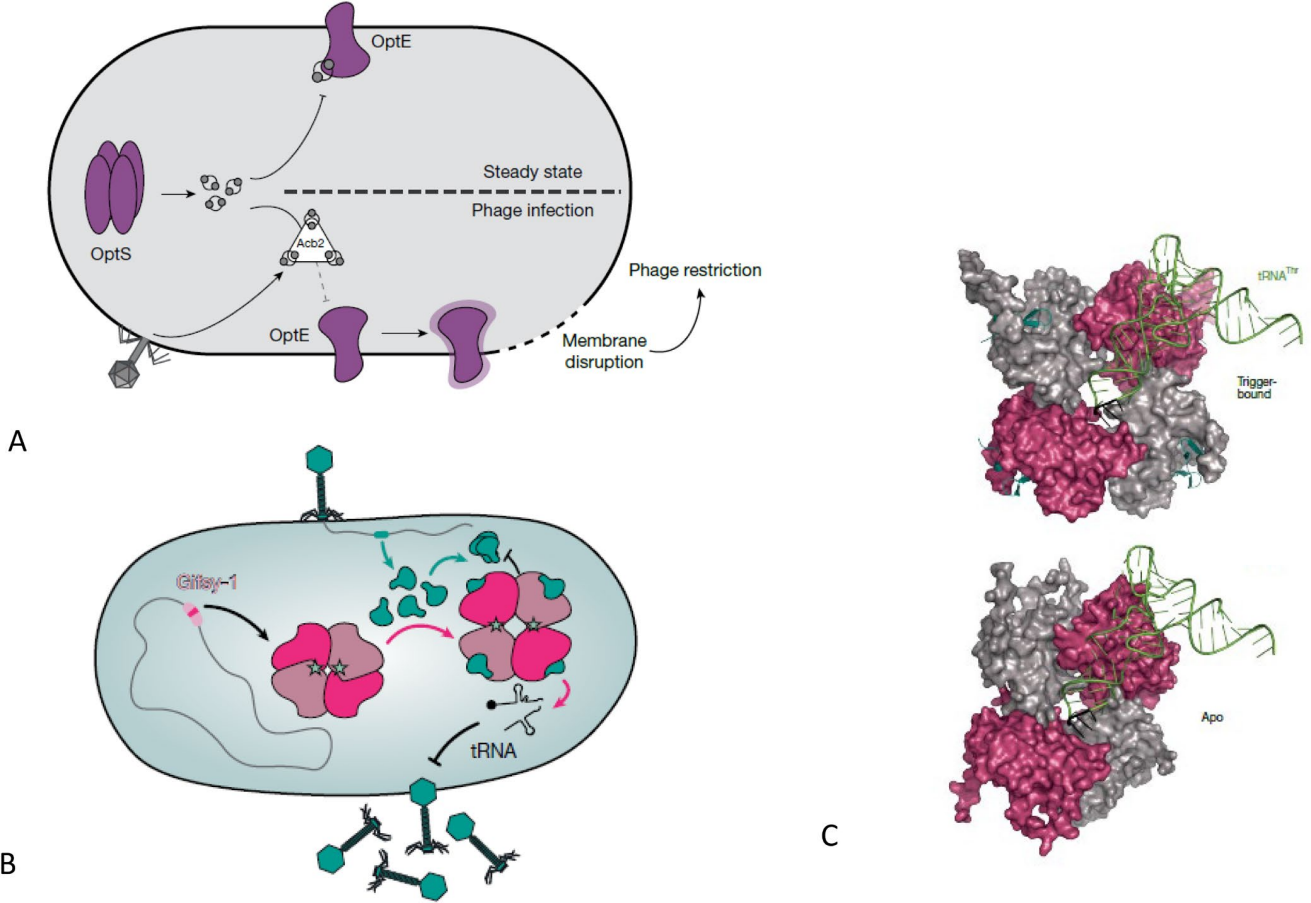
Models for the Panoptes and HepS APS. (A) Panoptes APS consists of OptS producing a cyclic dinucleotide shown in Fig. 1B. Its binding to the OptE effector, a transmembrane protein, prevents OptE activation. Phage T4 infection introduces the sponge protein Acb2 that adsorbs the cyclic dinucleotide. Absence of this signalling molecule activates OptE which disrupts the cell membrane and thus aborts the phage infection. (B) Prophage Gifsy‐1 produces the HepS protein. Upon binding of phage lambda tail protein J, the HepS protein undergoes a conformation change which opens the nuclease active site leading to the cleavage of tRNAThr in the anticodon loop. The stop of translation aborts the phage infection. (C) Structure of HepS in its open active conformation with associated tRNA (above) and in its closed enzymatically inactive conformation (below). Figure credit: Sullivan et al. (2025) (A) and Sargen et al. (2026) (B, C); under a Creative Commons Attribution License 4.0.

Other researchers described a two‐gene operon (*optSE*) from *Vibrio* consisting of a synthase S and an effector E containing transmembrane domains (Sullivan et al. 2025) (Figure 2A). When expressed in *E. coli* , OptSE mediated plaque reduction of phages including T4. The crystal structure of OptS revealed a tetrameric structure that aligns with the palm‐domain of Cas10 from type III CRISPR immune systems and that of a diguanylate cyclase. OptS indeed synthesizes a cyclic dinucleotide: 2′,3′‐c‐di‐AMP (Figure 1B), which associates with OptE, causing in the latter a conformational shift. Analysis of escape mutants of T4 elucidated the mechanism of this APS called Panoptes, after an all‐seeing giant of Greek mythology. T4 escapers showed a mutated T4vs.4 gene, also called *acb2* gene for anti‐CBASS, because it antagonizes the CBASS (Cyclic‐oligonucleotide based Antiphage Signalling System) immunity (for a review see Slavik and Kranzusch 2023; Hobbs and Kranzusch 2024). The phage protein acts as a nucleotide ‘sponge’, sequestering bacterial cyclic oligonucleotide signalling molecules. The OptS‐synthesized cyclic di‐nucleotide inhibited OptE. Expression of OptE in isolation caused cell death through membrane permeabilization. Also, other phage ‘sponge’ proteins activated the Panoptes system. Visibly, the Panoptes APS works by detecting the activity of phage counter‐defences. Phages are thus confronted with a dilemma: if they lose Acb2, they are susceptible to CBASS but are not subjected to Panoptes control (an option chosen by phage T2). In contrast, T4 has chosen to avoid CBASS by maintaining *acb*2 but then becomes susceptible to Panoptes inhibition. Panoptes is sensing phage infection through detecting immune evasion and apparently presents an old antiviral system that is in some form also found in eukaryotes.

A very similar two‐gene APS exists in *E. coli* (Doherty et al. 2025). The first gene was characterized as a minimal CRISPR polymerase (mCpol). Structural analysis revealed that mCpol adopts an active conformation without additional requirements such as RNA binding and constitutively produces 2′,3′‐c‐di‐AMP. The second gene is 2TMß, a transmembrane protein which multimerizes in the absence of cyclic‐di‐AMP, leading to the collapse of the inner membrane. mCpol constitutively synthesizes the cyclic dinucleotide that prevents toxic oligomerization of the transmembrane protein. This *E. coli* APS corresponds to the above‐described Panoptes system and also resembles a toxin‐antitoxin system.

Interestingly, Phage T4 encodes both a cyclic nucleotide degrader (T457B) and a cyclic nucleotide ‘sponge’ (T4vs.4). Expression of either protein was sufficient to induce cell death. Natural mutants of T4 that were insensitive to the Panoptes system lacked both genes. Notably, the mCpol‐2TMß operon in *E. coli* was located between the CBASS‐IIA and the Hachiman (see below) APS.

## 4 HepS: A Prophage Encoded tRNA Nuclease Activated by a Phage Tail Tip Protein

APS are frequently encoded on MGE such as prophages which protect the lysogenic bacterial host against phage superinfection. Deletion and mutational analysis in *Salmonella* prophage Gifsy‐1 identified an immunity function against lambdoid phages consisting of the single protein, HepS which contains a HEPN (higher eukaryotes and prokaryotes nucleotide‐binding) domain (Sargen et al. 2026) (Figure 2B). HepS homologues are widely distributed in bacilli and enterobacteria. Overexpression of HepS was not cytotoxic, suggesting that it is by default inactive. Structural analysis of HepS demonstrated a tetrameric protein complex. How HepS is activated was revealed by a phage λ escaper that contained a single amino acid change in the lambda phage tail tip protein J. The researchers demonstrated by crystallography that a peptide sequence of wildtype λ J protein bound to each HepS monomer as an allosteric activator. The phage tail tip peptide caused an opening of the tetramer which created an active enzymatic site for RNA nuclease activity cleaving specifically tRNA^Thr^ in the anti‐codon loop (Figure 2C). Notably, Gifsy‐1 and two further prophages of the Salmonella lysogen contained a single aa replacement in their protein J that failed to activate HepS.

## 5 Rip1 Pores: Combining Sensor and Effector Function Into a Single Protein

As shown by expression and deletion analyses, a *Pseudomonas aeruginosa* prophage contains an anti‐phage protein Rip1 for ring interacting pore 1, located between the small and large subunit prophage terminase genes (Patel et al. 2026). When the lysogen was superinfected with an indicator phage, the cell changed from the normal rod into a spherical form, suggesting that Rip1 activity disrupts inner membrane integrity. However, Rip1 expression on its own was not toxic for the cell; it apparently required a trigger from phage infection. Phage escapers carried mutations in the portal protein of *Pseudomonas* phages and in the small terminase (ST) protein of *E. coli* phages. Biochemical and cryo‐EM analysis demonstrated that phage ST proteins formed a ring consisting of 11 monomers, around which Rip1 forms a second ring composed of 12 monomers. The N‐terminal part of Rip1 formed an amphipathic helix structure while the C‐terminal half assured contact with the ST protein ring. A conspicuous pore structure was revealed going through Rip1 and ST ring structure resembling that of pore‐forming toxins. Mutants of ST which lost the capacity to oligomerize did not form the pore structure. The pore structures mediated dye release from liposomes, and microscopy showed the build‐up of holes in *E. coli* exposed to Rip1‐ST pores. The researchers concluded that Rip1 senses late products of phage morphogenesis and simultaneously forms a pore which prevents the orderly final step of phage maturation by prematurely lysing the infected cell. Bioinformatic analysis showed that Rip1 related genes were widely distributed in proteobacteria; most of them were found in prophage context.

## 6 KIWA: A Cell Membrane Associated Protein Complex Inhibiting Phage DNA Injection

**FIGURE 3:**
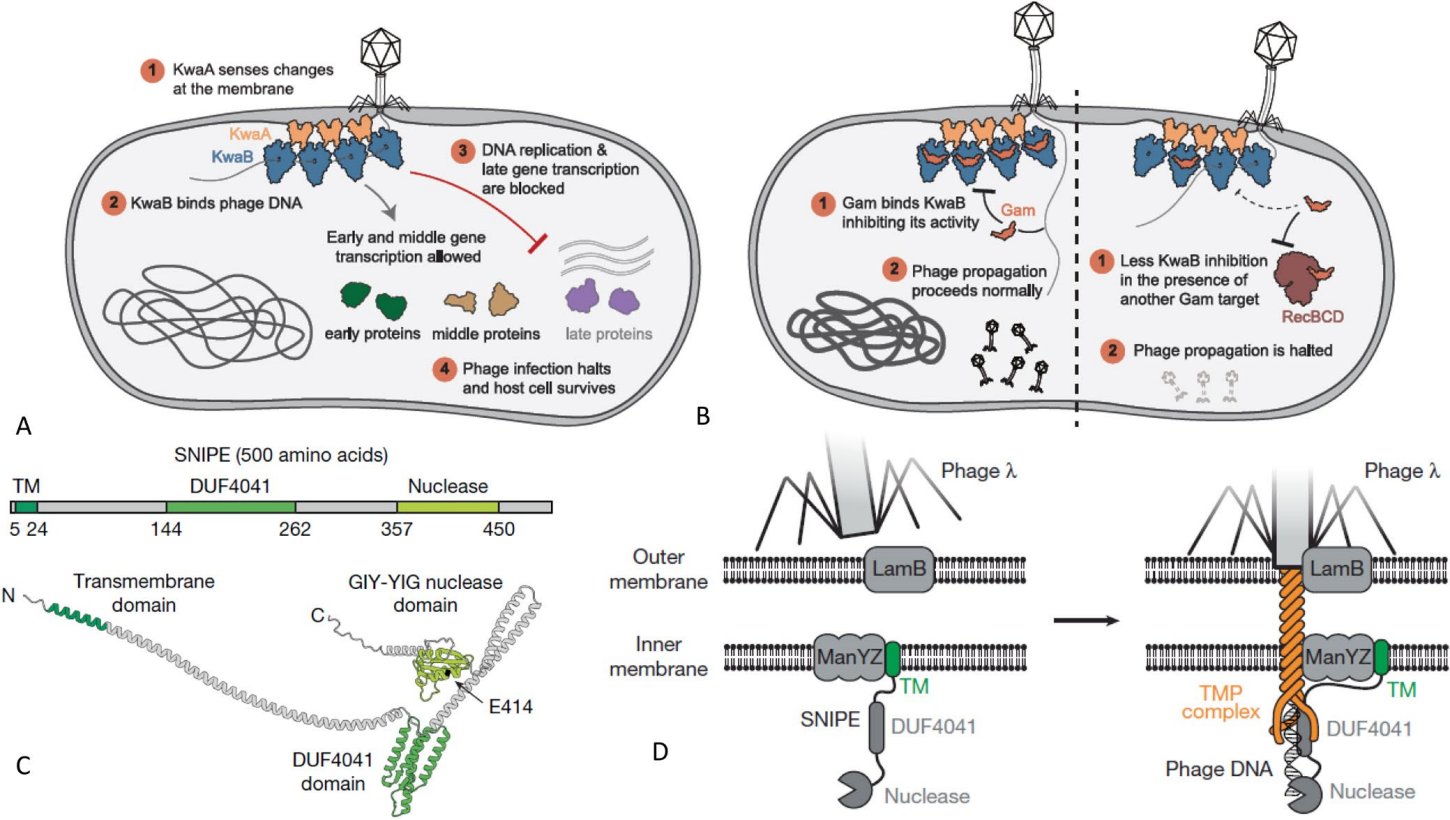
Models for KIWA and SNIPE APS action. (A) Stepwise model for the action of the KIWA APS as proposed by Zhang et al. (2025) leading to a block of late phage transcription which blocks phage replication. (B) Model developed by Zhang et al. (2025) how phage lambda Gam protein binds and inhibits KiwaB protein, counteracting the inhibition by KiwaAB. Phage infection can then proceed (left part). When a second RecBCD APS is introduced which also binds Gam, less Gam is available to inhibit KiwaB and phage inhibition is reconstituted (right part). (C) Domain organization (top) and structure prediction (bottom) for the antiviral protein from the SNIPE APS. (D) Model proposed by Saxton et al. (2026) how phage lambda adsorbs to its cell receptor LamB at the outer membrane and interacts with the host ManYZ protein in the inner membrane during the DNA injection process. The antiviral SNIPE protein associates with ManYZ which positions the protein for nuclease attack on phage DNA during entry into the cell. Figure credit: Zhang et al. (2025) (A); Saxton et al. (2026); under a Creative Commons Attribution License 4.0.

The Kiwa phage resistance system consisting of a two‐gene operon: KwaA, a four‐pass membrane protein and KwaB, of unknown function (Zhang et al. 2025) (Figure 3A). This phage resistance system is widely distributed among enterobacteria and bacilli and mostly located in the neighbourhood of mobile genetic elements. These researchers cloned the Kiwa system in *E. coli* where it conferred phage protection against Myo‐, Sipho‐ and Podophages. Cryo‐EM of the KwaAB complex showed a KwaA tetramer bound to four KwaB dimers that formed large fence‐like supercomplexes at the inner cell membrane. KwaA inserts into the membrane with a short extension into the periplasmic space. KwaB is fully located in the cytoplasm. Loss of either KwaA or KwaB abolished phage defence. Even at high infection load (MOI = 10), Kiwa prevents productive phage infection without killing the host. RNA sequencing of phage T2‐infected KIWA containing cells demonstrated a downregulation of late phage transcripts encoding structural proteins. KwaB structure predicts a positively changed central channel which could bind DNA. Electrophoretic mobility shift assays indeed demonstrated binding of both ssDNA and ds DNA from phage. Mutation of critical channel contact sites abolished defence and DNA binding. KwaB is activated by KwaAB complex formation and colocalizes to phage attachment sites at the cell poles where phage DNA injection occurs. T7 phage mutants that escape Kiwa defence were located to the phage tail fibre protein, suggesting that the periplasmic part of KwaA acts as the membrane sensor that then activates the DNA‐binding effector KwaB. KwaB phage DNA binding is compatible with early and middle phage transcription while late gene transcription is blocked. As a result phage infection halts and the infected cell survives.

Phages often encode proteins that counteract bacterial immunity. When testing such proteins, the researchers observed that the phage lambda protein Gam strongly inhibited Kiwa defence (Figure 3B). Gam is a DNA mimetic that binds KwaB similar to phage DNA and allows phage replication to proceed in cells with the Kiwa system. Lambda Gam also binds and inhibits the bacterial RecBCD system that degrades phage DNA. In the presence of both the Kiwa and the RecBCD systems, limiting Gam concentrations are binding to both Kiwa and RecBCD. Through this diluting effect, the anti‐anti‐defence efforts of the phage are spoiled. The researchers suggested that this effect is a reason for the frequent observation of multiple often adjacent APS in bacteria.

## 7 SNIPE: Destroying Phage DNA at Injection Through the Membrane

The APS SNIPE (for surface associated nuclease inhibiting phage entry) detects phage DNA injection through the inner bacterial membrane. It blocks phage λ infection at an early step, allowing cells to grow normally (Saxton et al. 2026). AlphaFold analysis predicted for SNIPE an N‐terminal end in the periplasmic space, a transmembrane domain and a cytoplasmic domain of unknown function (DUF). At the C‐terminus, a nuclease domain was predicted and confirmed experimentally (Figure 3C). Mutation and deletion analysis showed that both DUF and the nuclease are necessary for antiviral activity. Fluorescence microscopy localized SNIPE to the cell membrane; deletion of the transmembrane domain was toxic to the cell. The membrane location of SNIPE sequesters the nuclease from attacking host DNA. DUF has a positively charged surface that might facilitate DNA binding. Using marked lambda genomes in the classical Hershey‐Chase experiment, it was demonstrated that phage DNA is digested when it is injected into the cell. How does SNIPE get directed to the phage genome injection site? The researchers demonstrated that SNIPE associates with the mannose permease ManYZ. Phage lambda uses LamB in the outer membrane of *E. coli* as a cell receptor; ManYZ is located in the inner membrane and assists phage DNA injection. SNIPE association with ManYZ places the DNA binder DUF and the nuclease in the right place to destroy phage DNA upon entry into the cell (Figure 3D). SNIPE offers broad defence against many siphophages, sometimes independently of ManYZ. Interactome analysis revealed SNIPE association with the tail tape measure protein (TMP) of these phages. Bioinformatic analysis indicated that SNIPE antiviral proteins are widely distributed in different bacterial clades, but showed substantial sequence variability. However, SNIPE showed no antiviral activity against myoviruses and podoviruses which use distinct genome injection mechanisms.

## 8 Zorya: A Membrane Motor Senses Phage DNA Injection and Activates Nuclease Effectors

**FIGURE 4:**
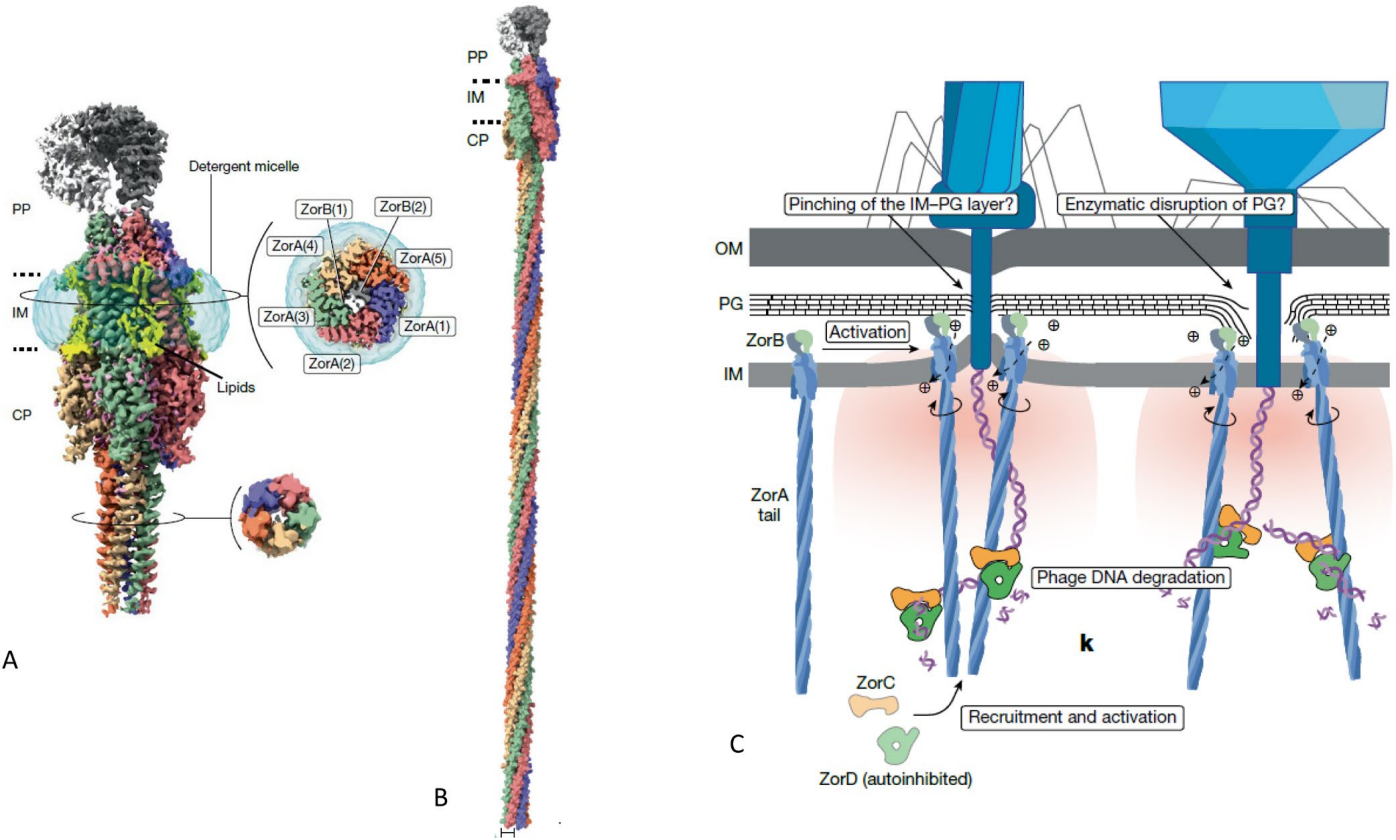
Model for Zorya APS action. Panel (A) shows the upper part of the Zorya APS that consists of a ZorA_5_B_2_heteropolymer reaching into the periplasmic space (PP), crossing the inner membrane (IM) and extending into the cytoplasm (CP). Lipids associated with the proton‐driven motor unit are indicated as well two horizontal cuts through the structure at the level of the inner membrane and in the cytoplasm with the positioning of the Zorya A and B protomers and the central channel indicated. Panel (B) displays the long cytoplasmic tail of the ZorAB complex cut short in Panel (A). Panel (C) displays the model for the action of the Zorya APS developed by Hu et al. (2025). The ZorAB complex is activated when it senses the entry of the phage DNA into the cell either through the pinching of the peptidoglycan (PG) layer or the pinching of the inner membrane (IM) by the intruding phage tail or the digestion of the PG by the phage tail. Activation induces proton flow (+) and circular motion of the ZorAB motor unit that leads to association with the ZorC,D subunits that mediate the degradation of the injected phage DNA. Figure credit: Hu et al. (2025) under a Creative Commons Attribution License 4.0.

Zorya APS are widespread in Gram‐negative bacteria. Type I Zorya protects against T1‐ and T7‐like phages, but not against T4‐ and T5‐like phages without compromising bacterial growth. As the SNIPE system, Zorya does not interfere with non‐phage foreign DNA invasion, such as plasmids. An international consortium investigated the complex molecular underpinnings of this antiviral system (Hu et al. 2025) (Figure 4). Zorya is a four‐gene operon (*zorABCD*). Recombinantly expressed ZorAB revealed, upon negative stain EM, a head domain attached to a long tail. Cryo‐EM and quantitative mass spectrometry resolved a ZorA_5_B_2_ structure, resembling rotary bacterial motors such as the flagellar stator unit. The ZorB dimer reaches into the periplasmic space and displays a peptidoglycan binding domain. In the transmembrane region, ZorB is surrounded by five ZorA units, and the complex extends with a 70 nm long tail structure into the cytoplasm. A water‐filled ion‐permeation pathway passes through the complex, suggesting that ZorA_5_B_2_ represents a proton‐driven motor. Mutational analysis revealed that all four Zor proteins are necessary for phage resistance. The data suggested that the ZorA_5_B_2_ structure is the sensor which transmits a signal to the ZorC,D effectors. Electrophoretic mobility shift assays demonstrated that ZorC binds dsDNA in a sequence‐independent way. The C‐terminal part of ZorD displays nuclease activity which is, however, inhibited by the N‐terminal part. This autoinhibition is abolished by a conformational change. Upon phage infection, cytosolic ZorC and D are recruited via the long tail to the ‘activated’ membrane‐associated ZorA,B complex. Fluorescence microscopy showed that phage DNA was still injected into the cell but rapidly degraded by the Zorya complex, assuring the survival of the cell. The authors developed a mechanistic model for Zorya action: when a phage injects its genome, it disturbs the bacterial envelope, pulling the inner membrane closer to the peptidoglycan layer. ZorA,B which diffuses laterally in the inner membrane can now contact the peptidoglycan via its periplasmic domain which activates its ‘motor’ unit leading to ion inflow and recruitment and activation of ZorC,D via its tail. With this construction layout, nuclease activity is only activated at the site of phage entry while host DNA is spared, allowing a sequence‐independent antiviral system.

## 9 Hachiman: A Helicase‐Nuclease Complex Destroying Phage and Host DNA After Scanning for DNA Damage

**FIGURE 5:**
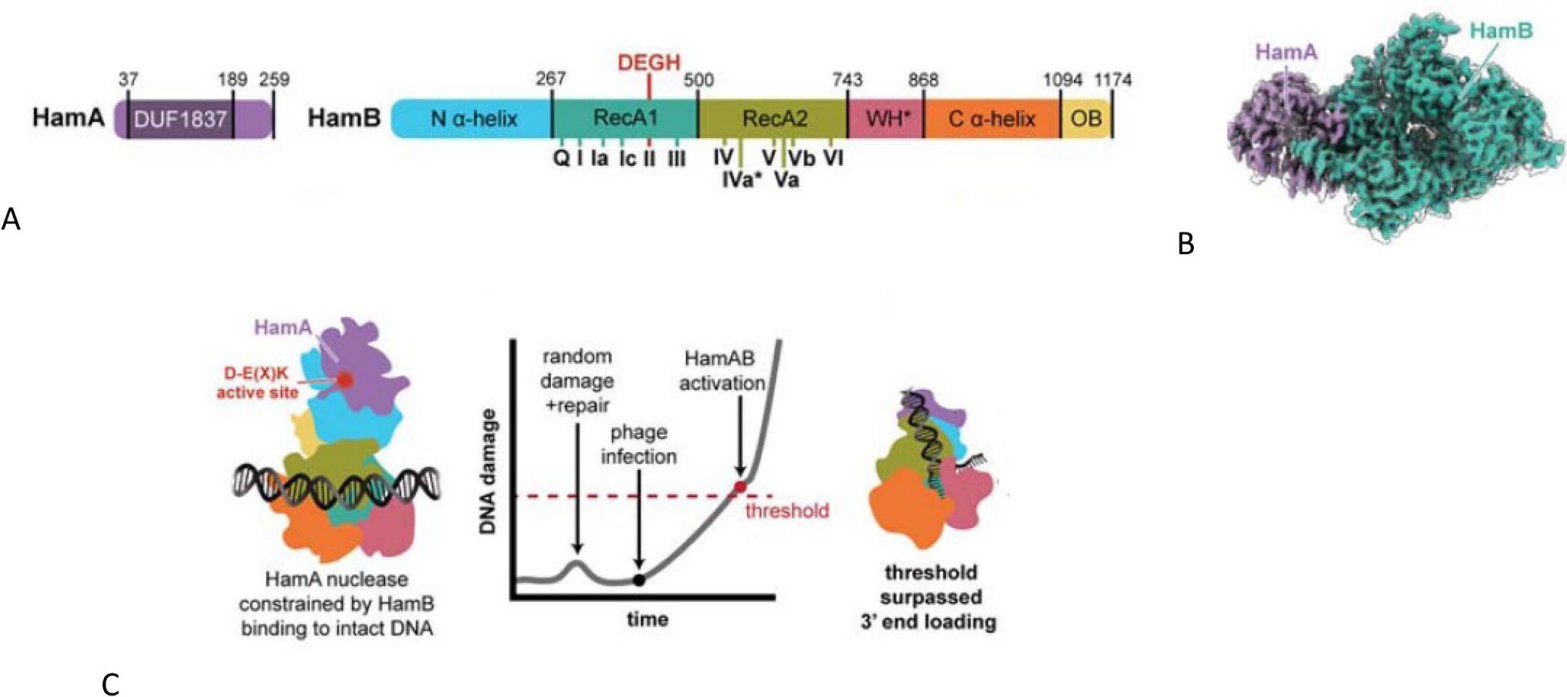
Model for Hachiman APS action. APS Hachiman consists of two proteins, a nuclease HamA that must be activated by a helicase HamB. Panel (A) shows the domain composition of the Hachiman proteins HamA (DUF, domain of unknown function) and HamB (RecA1, 2 Rec‐like helicase domains; OB oligonucleotide binding domain; DEGH, active site residues). Panel (B) shows the cryo‐electron microscopy structure of the 1:1 HamA : HamB complex. Panel (C), left, shows the structure of the HamA nuclease with its free active site and the dsDNA bound to the HamB helicase. The domain structure is colour‐coded according to Panel (A). Centre: HamAB surveys DNA in a scanning mode to monitor DNA damage. Damage is low in repair‐competent bacterial cells while DNA transactions in phage DNA replication create ssDNA overhangs and activate HamAB. Subsequently, DNA changes the position bringing it in contact with the nuclease active site as indicated at the right side. Figure credit: Tuck et al. (2024) under a Creative Commons Attribution License 4.0.

Analysis of the DefenseFinder database demonstrated that one‐third of APS are restriction/modification systems, followed by about 10% associated with helicases while the remaining half are represented by a diverse collection of defence systems. Tuck et al. (2024) analysed in molecular detail one of such helicase‐associated systems, Hachiman, identified in several *E. coli* strains (Figure 5A–C). The system consists of a two‐gene operon HamAB. Hachiman inhibited multiplication of various dsDNA phages at low MOI; ssRNA‐, ssDNA‐phages and T5 dsDNA phages were not affected. Hachiman represents an abortive infection (abi) type of resistance. HamB displays two helicase domains, a C‐terminal nucleic acid binding cleft and an N‐terminal binding interface with HamA. HamA shows a nuclease domain but must be activated by HamB to trigger this effector function. Cryo‐EM revealed a 1:1 heterodimer (HamA1:HamB1) complex. That HamB is a helicase was demonstrated experimentally by ATP‐dependent unwinding of a 15‐bp duplex with a 15‐nt 3′ overhang. In vitro, HamAB cleaves plasmid DNA followed by further DNA degradation. Recognition of a 3′ DNA overhang is important for immune activation. The researchers investigated Hachiman activity in phage infected cells by time course fluorescence microscopy. Degraded DNA begins to appear 10 min post infection (pi) and 50 min pi most cells are phantom cells, containing neither phage nor host DNA. Hachiman degrades phage and bacterial DNA without distinction. It is therefore important that the complex is inhibited in the absence of phage infection. The scientists demonstrated that Hachiman could be activated in the absence of phage infection when treating cells with antibiotics inducing DNA damage. Apparently, HamAB surveys DNA in a scanning mode to monitor DNA damage. Damage is low in repair‐competent bacterial cells while DNA transactions as obligatory steps in phage DNA replication activate HamAB. HamAB represents thus an antiviral system with broad phage coverage but at the expense of a low level of autoimmunity and efficient collateral host DNA degradation when phage infection is detected.

## 10 Recurrent Use of Coevolved Protease‐Nuclease Pairs in Divers APS

Tuck et al. (2026) also described a new APS consisting of a pair of co‐occurring genes. One gene predicts a nuclease followed by a trypsin‐like protease fused to the N terminus of a defence‐associated enzyme, for example to HamA from the Hachiman system. They cloned the HamMAB operon from *Pseudomonas* in *E. coli* which provided protection against Vequintaviruses. Deletion analysis demonstrated that all three genes are needed for phage resistance. They found that the protease domain fused to HamA cleaved the nuclease‐like HamM into three fragments which concomitantly activated a strong DNase function. The uncleaved HamM contains an insertion that blocks the active site. Proteolytic removal of the insertion freed the DNase activity. They concluded that HamM is a ‘gated’ pronuclease that acquired regulatory insertions responsive to a partner protease. The experiments indicated that upon damaged DNA sensing, HamB hydrolyzes ATP and triggers protease‐HamA, leading to HamA oligomerization and proteolytic cleavage of HamM, unmasking the nuclease active site and unleashing nuclease activity. Comparable nuclease‐protease pairs were also detected in other APS. Notable is the canu system from Klebsiella, KaeCanABC, which provides protection against T4 phage. CanC is again a nuclease whose active site is likely blocked by dual disordered insertions. CanA is a caspase family protease, structurally similar to a human paracaspase. T4 escape mutants map to the phage *dda* gene, a nonessential T4 helicase involved in phage DNA replication, repair and recombination. The cooperative nuclease‐protease pair apparently coevolved and was mobilized by lateral transfer to diverse APS as a regulatory element.

## 11 Hailong: A DNA‐Gated Ion Channel as a Phage Trap

Tan et al. (2025) described the mechanism of action for the Hailong APS. Hailong consists of a two gene operon: HalA, a transmembrane protein and HalB, a putative nucleotidyltransferase (NTase). This antiviral system was detected in 70 genera of gram‐positive and gram‐negative bacteria. The cloned HalB mutated in the NTase active site was toxic to the cell. The researchers suspected that HalB negatively regulates HalA activity. The crystal structure of HalB revealed a homotetramer protein covalently associated with an oligodeoxyadenylate (ODA). HalB processively synthesized long poly‐dA chains that are probably transformed into ODA by cellular enzymes. The ODA de novo synthesis is template‐independent and primed by a tyrosine hydroxyl group of HalB. In the absence of HalB activity the cloned HalA membrane protein is also toxic to the cell. Cryo‐EM analysis showed a homo‐tetrameric HalA protein with a transmembrane domain, a long central channel and a cytoplasmic crown‐like extension. Five nt‐long ODA molecules bound at each inter‐subunit interface. Modelling predicted that the central channel opens in the absence of ODA binding. The researchers provided experimental evidence that HalA functions as a DNA‐gated ion channel. HalA expressed without HalB induces membrane depolarization with Na^+^ inflow. These data identified HalA as effector and HalB as negative regulator of this antiviral system. What is the molecular cue responsible for defence activation in vivo? An answer came from the analysis of phage SECφ4 (a tailed Dhillonvirus) escaper mutations located in a single phage gene encoding an enzyme with a promiscuous 5′‐3′ DNA exonuclease activity which also degrades ODA. Expression of this single phage protein could induce the Hailong antiviral system. Contrary to antiviral systems creating an oligonucleotide second messenger to activate the system, an oligonucleotide is here synthesized constitutively and used as a trap for an infecting phage.

## 12 DRT2: Rolling Circle Reverse Transcription Leading to an Endless Protein Inducing Cell Dormancy

**FIGURE 6:**
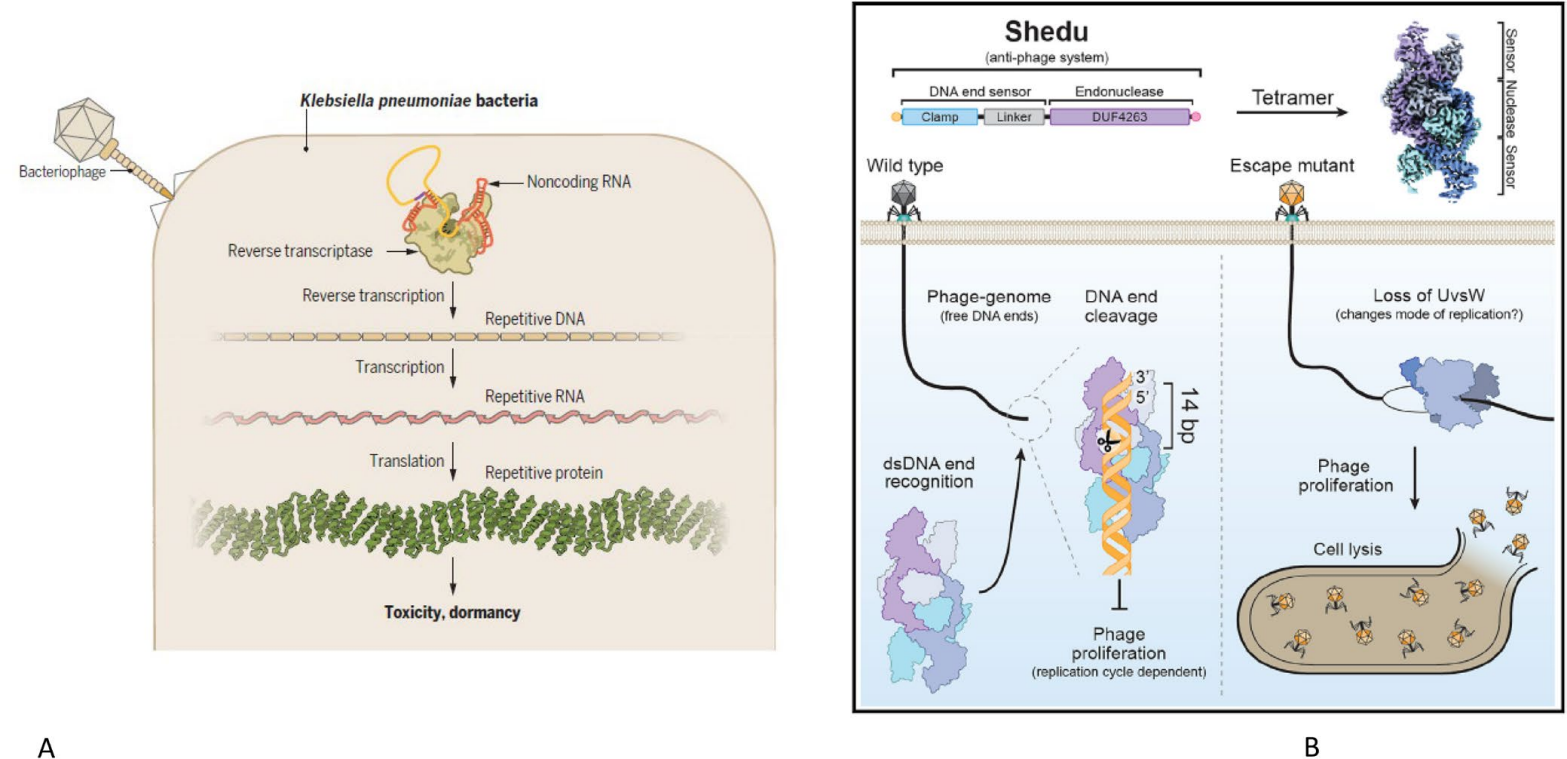
Model for DRT2 and Shedu APS action. Panel (A) Repetitive gene synthesis by DRT2 reverse transcriptase. Upon phage infection, the reverse transcriptase synthesizes a repetitive DNA molecule, which is transcribed into a repetitive RNA, which is translated into a repetitive protein. The protein is toxic and triggers cellular dormancy, inhibiting phage propagation. Panel (B) The Shedu APS consists of a single protein composed of a DNA end sensor, displaying a clamp structure, a linker domain and an endonuclease domain. It forms a homo‐tetramer with sensor clamps at both ends and a central nuclease region. Upon detection of dsDNA ends, it cleaves at a fixed distance from the bound DNA end. Phages escape through a mutation in the uvsW gene, a branch‐migrating dsDNA helicase. After loss of UvsW, phages switch to an alternative mode of DNA replication which is no longer subject to Shedu control. Figure credit: Wilkinson et al. (2024) and Loeff et al. (2025) under a Creative Commons Attribution License 4.0.

The defence‐associated reverse transcriptase (DRT) system 2 consists of a single ORF encoding a reverse transcriptase (RT) and an upstream non‐coding RNA (ncRNA). Tang et al. (2024) studied this antiviral system from *Klebsiella pneumoniae* (Figure 6A). They identified RNA and cDNA of opposite strandedness associated with the immunoprecipitated RT from uninfected cells. The sequences of both RNA and cDNA corresponded to the ncRNA genome region. Upon phage infection, they observed a strong induction of cDNA copies and second strand DNA synthesis. The ncRNA has a complex secondary structure consisting of seven stem‐loop (SL) elements. Mutations in four SL regions resulted in loss of phage defence. The researchers observed that the RT switched from the end of the template region back to the start, resulting in concatemeric cDNA repeat products with a uniform head‐to‐tail junction. The scientists deduced that the nascent DNA strand dissociates from its RNA template and reanneals with the complementary sequence element in the RNA. Template jumping initiates a subsequent round of reverse transcription, with concatenation of one cDNA repeat to the next and incorporation of one additional base at the repeat junction, ultimately leading to long concatemeric cDNA (ccDNA) products. Repeat lengths of up to 40 were determined by Nanopore sequencing. As the process resembles rolling circle DNA replication, the researchers called it ‘rolling circle reverse transcription’ (RCRT). The single‐stranded concatemeric cDNA (ccDNA) was then efficiently converted into dsDNA in a phage‐ and RT‐dependent manner. From this dsDNA the host RNA polymerase copies abundant RNA shortly after phage infection. The RNA contains a ribosome binding site and a long open reading frame without stop codons repeating 40 sense codons. The lack of any in‐frame stop codon results in a *neo* (nearly endless ORF). The introduction of a nonsense codon into ncRNA abolished phage resistance. By proteomics analysis using tandem mass spectrometry analysis, neo‐derived peptides were only detected in phage‐infected cells that expressed wildtype RT. Phage proteins were diminished while two host proteins were increased, namely ArfA (associated with ribosome stress) and RMF (associated with ribosome hibernation). The researchers suggested that RMF might induce a kind of cell dormancy stopping phage propagation. Even at high MOI, the dormant cell remained viable. Candidates for ncRNA concatemers and neo protein synthesis were widely distributed among bacteria. Another US lab investigated the same system and arrived at comparable results only differing in details (Wilkinson et al. 2024). They note conceptual similarities between prokaryotic DRT2 and eukaryotic telomerase, which they attributed to convergent evolution.

## 13 DRT9: Reverse Transcribed Poly‐dA as Phage Sensor (And as Phage DNA Decoy?)

Two groups investigated the mechanism of action for DRT9. When cloned and expressed in *E. coli* , DRT9 defended against T4‐ and T5‐like phages. At low MOI with T5, cell growth continued; at high MOI programmed cell death was initiated. Mutation in the active site of the RT abolished phage inhibition. Tang et al. (2025) initially suspected that DRT9 would reverse transcribe the ncRNA into DNA. However, no cDNA corresponding to ncRNA was detected. Southern blotting identified the synthesis of poly‐dA tracts of up to 10,000 nucleotides length. Further biochemical analyses identified the template and primer for this peculiar poly‐dA synthesis. The template is a small tract of 4 uridines in the ncRNA, located in a loop between numerous base‐paired stem structures. Mutating the U4 bases abolished the phage defence. Apparently DRT9 repetitively spools out tracts of A4 into a long dA homopolymer. No nucleic acid primer is used for this reaction. Mobility shifts of the RT indicated that the synthesized ssDNA remained covalently linked to the enzyme. Mutation analysis demonstrated that the hydroxyl group of two tyrosine residues served as protein primer. Cryo‐EM studies revealed a homo‐hexamer for the RT consisting of trimers of dimers. The RT showed the usual finger, thumb and palm structure; all three elements contacted the associated ncRNA. The U4 was close to the active site of the enzyme. DRT9 is inactive in uninfected cells. There remained a discrepancy: in vitro poly‐dA synthesis is constitutive while in vivo poly‐dA synthesis is strictly phage dependent. How is this anti‐phage defence system regulated in bacteria? Part of the answer was again provided by escape mutants of phage T5 able to grow in the presence of DRT9. All deletion mutants of T5 escapers overlapped a promotor of a six‐gene phage operon. Mutation analysis revealed that a single T5 gene, gp58, prevented cell growth when co‐expressed with DRT9. The phage gene shares similarity with a primosomal protein that restarts damaged replication forks. Co‐immunoprecipitation revealed that gp58 indeed interacted with DRT9 and its poly‐dA, probably at the 3′ end. Another cue came from further in vitro experiments. The poly‐dA strand was efficiently degraded by the host exonuclease ExoI while the presence of the phage gp58 protected poly‐dA from degradation. However, how this process leads to programmed cell death remains undefined. Co‐immunoprecipitation proteomics led to three proteins associated with DRT9, namely a T5 single‐stranded DNA binding protein and two host‐encoded nucleoid‐associated proteins, H‐NS and StpA. These proteins might thus be involved in mediating cell death as an abortive infection mechanism for DRT9.

Chinese researchers investigated the DRT9 independently (Song et al. 2025). While the molecular data leading to poly‐dA synthesis are comparable, the observations with respect to the mechanism of phage inhibition and impact on cell growth differ. Escape mutants showed frameshifts or deletions in NrdB for T4 and NrdA for T5. NrdA and NrdB are class I ribonucleotide reductases that convert ribonucleotides into deoxyribonucleotides. This conversion is needed to feed phage DNA synthesis during infection. dNTP concentrations are substantially increased during T4 infection. Notably, T4 NrdAB expression alone can activate the DRT9 system leading to the synthesis of long poly‐dA chains. However, long poly(A)‐rich single‐stranded cDNA were not toxic to the host cell. This concurs with their observation that all infected cells eventually recovered from phage‐induced growth arrest. They then asked how DRT9‐synthesized single‐stranded cDNA contributes to antiphage defence. Depleting the intracellular dATP pool by poly‐dA synthesis was not inhibitory since the remaining dATP in DRT9‐expressing cells was sufficient for phage replication. Electrophoretic mobility shift assays demonstrated that T4 phage single‐stranded DNA binding (SSB) protein associated in vitro with poly‐dA and SSB bound poly‐dA in vivo. The Chinese scientists thus came up with a different model: In the absence of phage infection, DRT9 synthesizes only small amounts of poly‐dA partly due to low pools of dATP in the cell. Upon T4 infection the pool of cellular dATP increases substantially concomitantly increasing the synthesis of poly‐dA. The linear poly‐dA is an excellent target for T4 phage SSB binding. Since T4 SSB (gp32) is a crucial protein for T4 DNA replication, repair and recombination, sequestering SSB to DRT9 poly‐dA blocks T4 infection. Host cells resume growth after phage clearance. However, the data do not explain how DRT9 mediates transient cell growth arrest.

## 14 DRT3: Mixed Templated and Untemplated Repeat DNA Synthesis Becomes Cytotoxic in Presence of a Phage Protein

The DRT3 antiphage system consists of three genes: two genes encode the reverse transcriptases DRT3a and DRT3b followed by ncRNA (Deng et al. 2026). The two RT display congruent phylogenetic trees suggesting co‐evolution. The system is widely distributed across 20 bacterial phyla and protects *E. coli* against phages T1, T4 and T5, representing molecularly very distinct phage groups. Sequencing of cell lysates revealed a phage‐independent production of double‐stranded DNA (dsDNA) consisting of poly(GT/AC) dinucleotide repeats. In Cryo‐EM analysis, the DRT3 complex from *E. coli* (EcDRT3) consists of six copies each of Drt3a, Drt3b and the ncRNA of 130 nt length in a 1:1:1 stoichiometric ratio. When the complex is incubated with dNTP in vitro, the DRT3a subunit showed increases in molecular weight indicating covalent linkage of the synthesized ssDNA chain growing up to several kb length, which could only be liberated after protease treatment. The complex yielded both poly(GT) and poly(AC) reads, the complementary strands annealed and the resulting duplex DNA product was positioned around the exterior of the complex. The researchers demonstrated that DRT3a synthesizes poly(GT) ssDNA templated by ncRNA. The A^108^CACAC^113^motif of ncRNA was located at the active site of DRT3a. Mutating either A or C in positions 108 and 109 abolished phage resistance. A “zipper head” of ncRNA was proposed to dissociate the product–template duplex following each cycle of dinucleotide addition. In contrast, DRT3b synthesizes poly(AC) ssDNA de novo without a nucleic acid template. Two aa side chains from DRT3a mimic a templating nucleobase, forming hydrogen bonds assuring the dinucleotide specificity in the ssDNA product. Mutating the two aa positions abolished both anti‐phage defence and poly(GT/AC) dsDNA synthesis. While the astonishing enzymatic activities of these two RT were studied in molecular detail, it remained less clear how the poly(GT/AC) dsDNA mediates phage resistance. Mutations in T1 escaper phages were located in the uncharacterized, but conserved ST61 gene of T1. Co‐expressing ST61 and EcDRT3 was constitutively toxic in *E. coli* , suggesting that the phage protein triggers a cytolytic program mediated by DRT3 ds repeat DNA, but the mode of action remained undefined.

## 15 Retron‐Septu System ‘Arrest‐And‐Release’ Mechanism for a Nuclease

The Retron Ec83‐Septu operon confers phage resistance and consists of four genes: an ncRNA, a reverse transcriptase RT, PtuA containing an ATPase domain and PtuB with an endonuclease domain. Details of its mechanistic action were described recently (Wang, Rish, et al. 2025). The ncRNA is transcribed into an RNA consisting of two flanking terminal inverted repeats embracing an msrRNA (multicopy ssRNA) and msdRNA (multi‐copy ssDNA) region. The msdRNA region is reverse transcribed into msdDNA by the cognate retron, primed by a conserved guanine residue near the inverted repeat site. When reaching an RNA region with secondary structure elements, reverse transcription stops and the cDNA is latched to the remaining msrRNA creating a shorter hybrid RNA–DNA molecule after nuclease clearing. All four elements of the Retron Ec83‐Septu operon form a large complex consisting of a central tetramer of PtuA, PtuB monomers on each side and RT on the top. The hybrid RNA–DNA resembles a golf club. The RNA head wraps around the RT, the RNA–DNA duplex is in the cleft of RT, while the long ssDNA stalk runs through the central pore formed by the PtuA hexamer. Mutating PtuA sites that interact with the DNA stalk abolish phage resistance. Mutating the active‐site residues of PtuB likewise abolishes phage resistance. The Retron‐Septu complex is glued together by RNA–DNA‐protein and protein–protein interactions. However, the complex showed no nuclease activity. The PtuAB subcomplex, but not PtuA or PtuB individually, degraded ssDNA. In the absence of the Retron‐Septu system, T5 phage degrades bacterial DNA within 30 min after infection and started at 60 min pi to package newly synthesized phage DNA into progeny phage particles. In the presence of the Retron‐Septu system, host DNA is still degraded by phage T5 infection, but progeny phage DNA packaging is no longer observed. Based on their experiments, the researchers developed a model where a phage enzyme, possibly exonuclease D15, degrades the msrDNA which disintegrates the autoinhibited Retron‐Septu complex into two PtuAB subcomplexes and RT. The PtuAB complexes display now nuclease activity which cleaves ssDNA from phage and thus stops phage replication. In their model, msdDNA is both a central negative regulator that locks the system in an inactive conformation and a phage sensor and a molecular switch that activates the nucleolytic activity of PtuAB. The researchers noted that this regulatory logic mirrors that of toxin‐antitoxin systems.

## 16 Superinfection Exclusion: Receptor Blocking for Heterologous and Homologous Phages

Taylor et al. (2025) studied *Pseudomonas* prophage JBD26 which encodes a protein that confers strong resistance against *Pseudomonas* phages that use type IV pili for infection. Bacterial two‐hybrid assays using a library of proteins involved in the morphogenesis of pili demonstrated interaction of this highly expressed prophage protein with PilZ. The prophage protein was therefore called Zip for PilZ‐interacting protein. Deletion of *pilZ* prevented type IV pilus formation. Conversely, overexpression of PilZ in the lysogen increased phage susceptibility. The researchers demonstrated that Zip does not block pilus assembly but modulates it by shortening pili at low cell density and eliminating them in a large fraction of cells at high density. LasR, the principal regulator of quorum sensing, regulates *zip* expression from the prophage. *P. aeruginosa* prophages thus exploit host‐cell quorum sensing to regulate anti‐phage defence. A lysogen deleted for zip showed in overnight cultures much lower prophage titers in the supernatant. Apparently, Zip expression prevented non‐productive reinfection of the lysogen by the released prophages (‘anti‐Kronos effect’). They showed that anti‐Kronos genes are also found among other *Pseudomonas* phages, for example, phage JBD44, which uses LPS as cell receptor and encodes genes that acetylate the bacterial O‐antigen, thus preventing self‐adsorption of the released phage to the lysogen. Likewise, *Salmonella* phage ε15 mediates serotype conversion of its LPS receptor. Furthermore, *E. coli* phage HK97 encodes a membrane‐localized protein that blocks phage DNA entry.

## 17 Shedu APS: Single Protein Recognizing and Cleaving Free Ends of Linear DNA

The Shedu system EcSduA protects *E. coli* against T‐even phages without inducing programmed cell death (Loeff et al. 2025) (Figure 6B). Shedu is a single antiviral protein forming a homotetramer. The complex consists of a dimer of dimers where two N‐terminal domains form a clamp that binds DNA ends which is connected via a flexible linker to a C‐terminal nuclease domain. Expression of the C‐terminal nuclease was toxic to the cell. In vitro, EcSduA cleaves dsDNA. Substitution of active site residues abolished dsDNA cleavage and phage inhibition. Structural analysis of EcSduA with and without a bound 40‐nt dsDNA revealed that the dsDNA extends from the N‐terminal clamp to the center of the EcSduA tetramer containing the nuclease active site. Binding did not depend on a specific sequence and resulted in a nicking of the DNA placed over the active site, resulting in a 13‐nt cleavage product. Linear DNA was a substrate while circular plasmid DNA was not cleaved. Phage T6 escapers contained mutations in the phage UvsW protein. Introducing a stop codon into the gene rendered T6 insensitive to the Shedu system. UvsW functions together with recombination mediator proteins UvsX and UvsY in homology‐directed DNA repair and recombination‐dependent replication of T‐even phages which proceeds via linear DNA elements. Shedu thus detects pathogen‐associated molecular patterns (PAMPs), here linear DNA created during phage replication which does not occur during bacterial replication.

## 18 CBASS: Versatile APS With Modular Organization and Links to Mammalian Innate Immunity Systems

**FIGURE 7:**
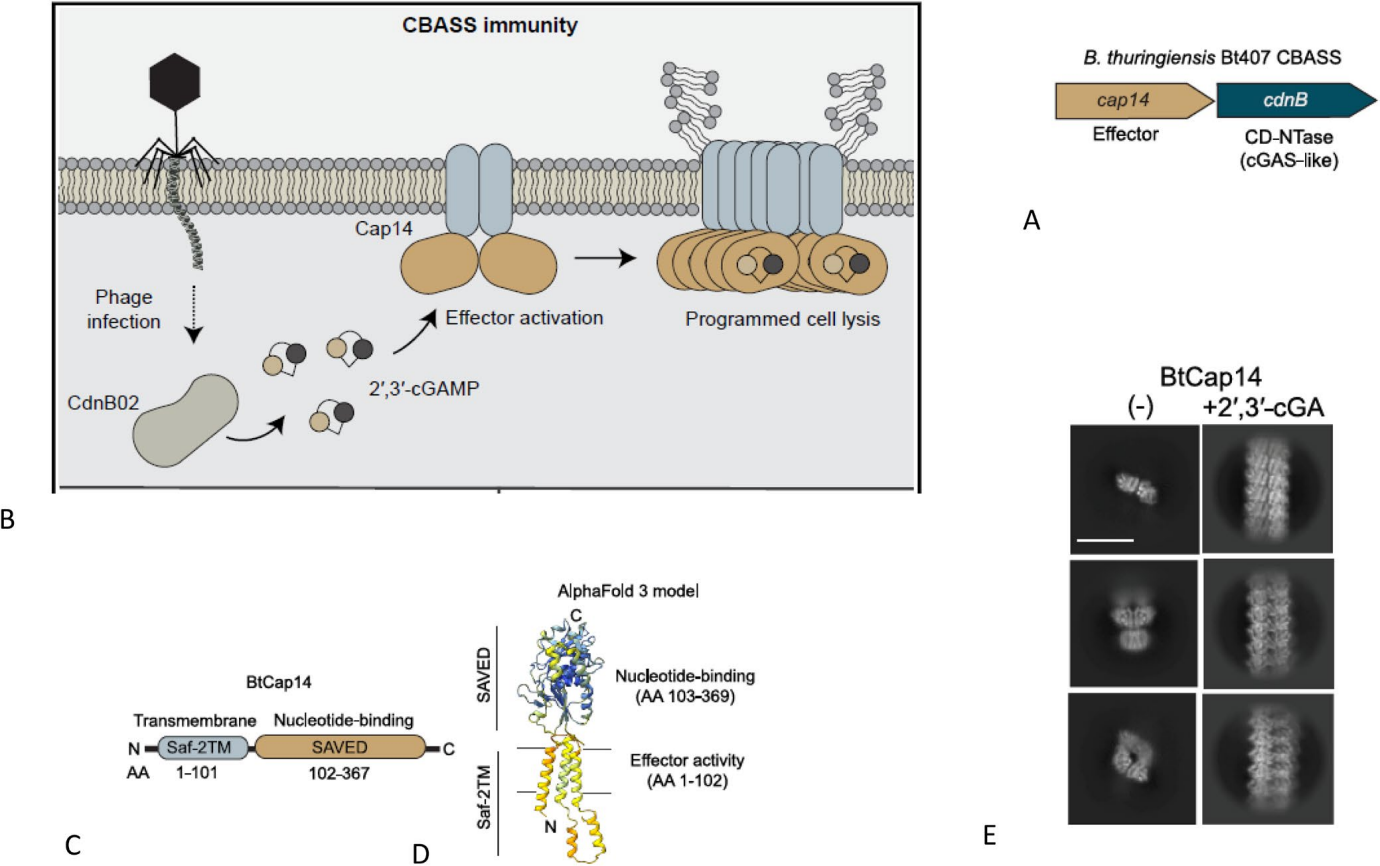
Mechanism of action for the CdnB‐Cap14 CBASS system. Panel (A) The type I CBASS operon from *B. thuringiensis* Bt407 consists of two genes, the cap14 effector and the cdnB sensor, a cGAS‐like CD‐NTase. Panel (B) presents a model for action. The CdnB enzyme synthesizes 2′,3′‐cGAMP in response to phage which activates the Cap14 effector to form filaments by oligomerization that abort phage infection via cell lysis. Panel (C) depicts the domain structure of Cap14 with an N‐terminal transmembrane Saf‐two transmembrane domain and a C‐terminal SAVED domain. Panel (D) shows a structure prediction for Cap14 with the nucleotide‐binding SAVED domain and the Saf‐2TM domain associated with effector activity. Panel (E) shows cryo‐EM pictures of Cap14 in the absence and presence of 2′,3′‐cGAMP signal which induces filament formation. Figure credit: Tak et al. (2026) under a Creative Commons Attribution License 4.0.

Cyclic‐oligonucleotide‐based anti‐phage signalling systems (CBASS) are widespread in bacteria. The system comprises a CD‐NTase (for cGAS/DncV‐like nucleotidyltransferase) which senses phage infection and synthesizes cyclic di‐ or oligonucleotide messengers that bind Cap (for CD‐NTase associated protein), an antiviral effector. Cap effectors are phospholipases, nucleases, NADases, or most frequently proteins with transmembrane (TM) segments. In the latter effector group, TM is associated with a SAVED domain. Such a system from *Bacillus thuringiensis* was studied by Gao et al. (2026). In the absence of phage infection, TM‐SAVED is an inactive monomer. Upon phage infection, CD‐NTase detects DNA and synthesizes 2′3′‐cGAMP (Figure 1F) which binds to SAVED. This leads over a transient TM‐SAVED dimer formation to a higher order filament formation. In the filaments a hairpin motif located between the TM segments rearranges. The protein forms a parallel array and creates an opposing hydrophilic and hydrophobic surface area in the membrane which leads to membrane bending and a vertical shearing of the lipid bilayer opening a fissure distinct from both classical pore formation and channel mediated permeabilization. These linear pores open 60 min after phage infection leading to cell death before phage maturation could finish, needing 90 min. Notably, bacterial CD‐NTase synthesizes 2′3′‐cGAMP with mammalian cGAS‐like enzymatic properties of innate immunity systems. Tak et al. (2026) reported comparable observations for Cap14 protein (Figure 7) and showed by electrophysiology that the pores become nonspecifically permeable to ions and water flow. They suggested that the SAVED domains are coordinating ligand‐dependent oligomerization to cluster effector domains independent of their downstream biochemical activity. To prove this point they constructed a functional chimera by swapping the transmembrane domain of Cap14 with a nuclease domain. They propose that such swapping occurs naturally in bacteria which would enlarge anti‐phage functions by modular exchanges.

Filament formation is also crucial for a CBASS having a phospholipase as effector protein. The CdnE senses phage infection by an unknown mechanism and synthesizes cUA as signalling messenger. The CapE phospholipase is present as a catalytically inactive dimer. Upon binding the cUA signal CapE dimers oligomerizes and form filaments on the cell membrane. This conformational change activates the phospholipase and mediates the destruction of the cell membrane. This early cell death aborts the phage replication cycle (Wang, Li, et al. 2025).

CBASS occurs in many forms, one peculiar variant repurposes enzymes involved in tRNA modification. The 7‐deazaguanine (carbon substitutes the N7 nitrogen) nucleobase named queuine (Q) is synthesized in bacteria and post‐transcriptionally incorporated into the anticodon loop of specific tRNAs. 7‐deazaguanines are installed into tRNA by the protein tRNA‐guanine transglycosylase (TGT). Surprisingly, Q biosynthetic genes have been recruited into APS such as type IV CBASS encoding the ancillary proteins Cap9 and Cap10, both of which are essential for phage defence and which share homology with Q biosynthetic enzymes QueC and TGT, respectively (Wassarman et al. 2025). In this APS, Cap10 forms a 2:2 heterotetramer complex with CD‐NTase CdnD. The CdnD N terminus is conjugated to a 7‐deazaguanine nucleobase catalysed by Cap9. Both Cap9 and Cap10 are essential for phage defence. The researchers suggested for this posttranslational protein modification a pathway that parallels the ubiquitin‐like conjugation systems found in type II CBASS APS. Filament formation activating effector function is a widely used principle of APS which will be illustrated by two APS.

## 19 Argonaute: Another Modular APS With Filamentation and Relatedness to Eukaryotic Systems

**FIGURE 8:**
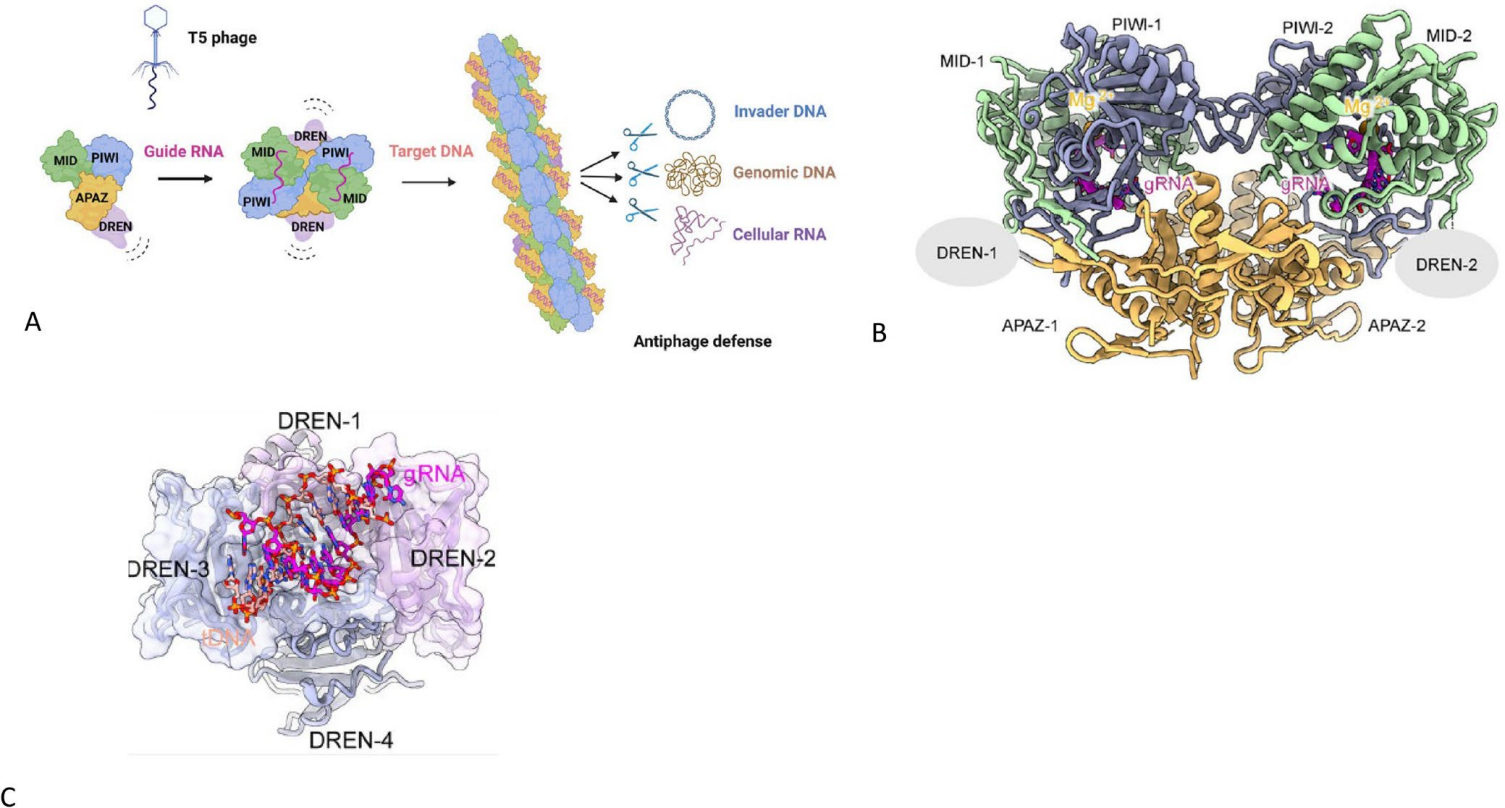
Mechanism of prokaryotic Argonaute SPARDA action. Panel (A) SPARDA comprises a short pAgo protein with MID and PIWI domains and the cognate DREN nuclease‐APAZ protein which can assemble into a heterodimer. The SPARDA heterodimer recognizes the guide RNA (gRNA), the heterodimer becomes a dimer and the recognition of target DNA (tDNA) by gRNA stimulates the filamentation of SPARDA, thereby activating nuclease activity by means of the tetramerization of the DREN domain. The DREN nuclease cleaves invader DNA, genomic DNA and cellular RNA. Panel (B) Shows the cryo‐EM structure of the gRNA‐bound SPARDA complex with the localization of MID and PIWI domains of pAgo and the DREN nuclease‐APAZ domains of the cognate protein. Panel (C) Depicts the cryo‐EM structure of the gRNA–tDNA duplex surrounded by the DREN nuclease tetramer from the filament structure. Figure credit: Zhang, Jiang, et al. (2026) under a Creative Commons Attribution License 4.0.

Prokaryotic Argonaute (pAgo) systems are smaller than eukaryotic Argonautes (eAgo). While eAgos display a four‐domain structure (N, PAZ, MID and PIWI) where MID and PAZ domains bind guide RNA (gRNA) and target DNA (tDNA) and the PIWI domain cleaves the tDNA, pAgo contain only two domains, MID and PIWI. In these systems, the PIWI domain is catalytically inactive. In APS, pAgo are associated with a second gene encoding an APAZ domain fused to a DREN nuclease domain (‘SPARDA’), forming a heterodimer (Zhang, Jiang, et al. 2026). Other pAgo utilize NADase effector domains (‘SPARTA, SPARSA’). In SPARDA pAgo, the MID/PIWI: DREN/APAZ proteins form a1:1 heterodimer and binds the guide RNA, which leads to a 2:2 heterodimer formation. The subsequent recognition of tDNA promotes filament formation, which activates nuclease activity through tetramerization of the DREN domain. SPARDA shows then nuclease activity that non‐specifically cleaves a wide range of nucleic acids aborting phage multiplication (Figure 8).

## 20 Cat1 Filaments Degrade NAD ^+^

**FIGURE 9:**
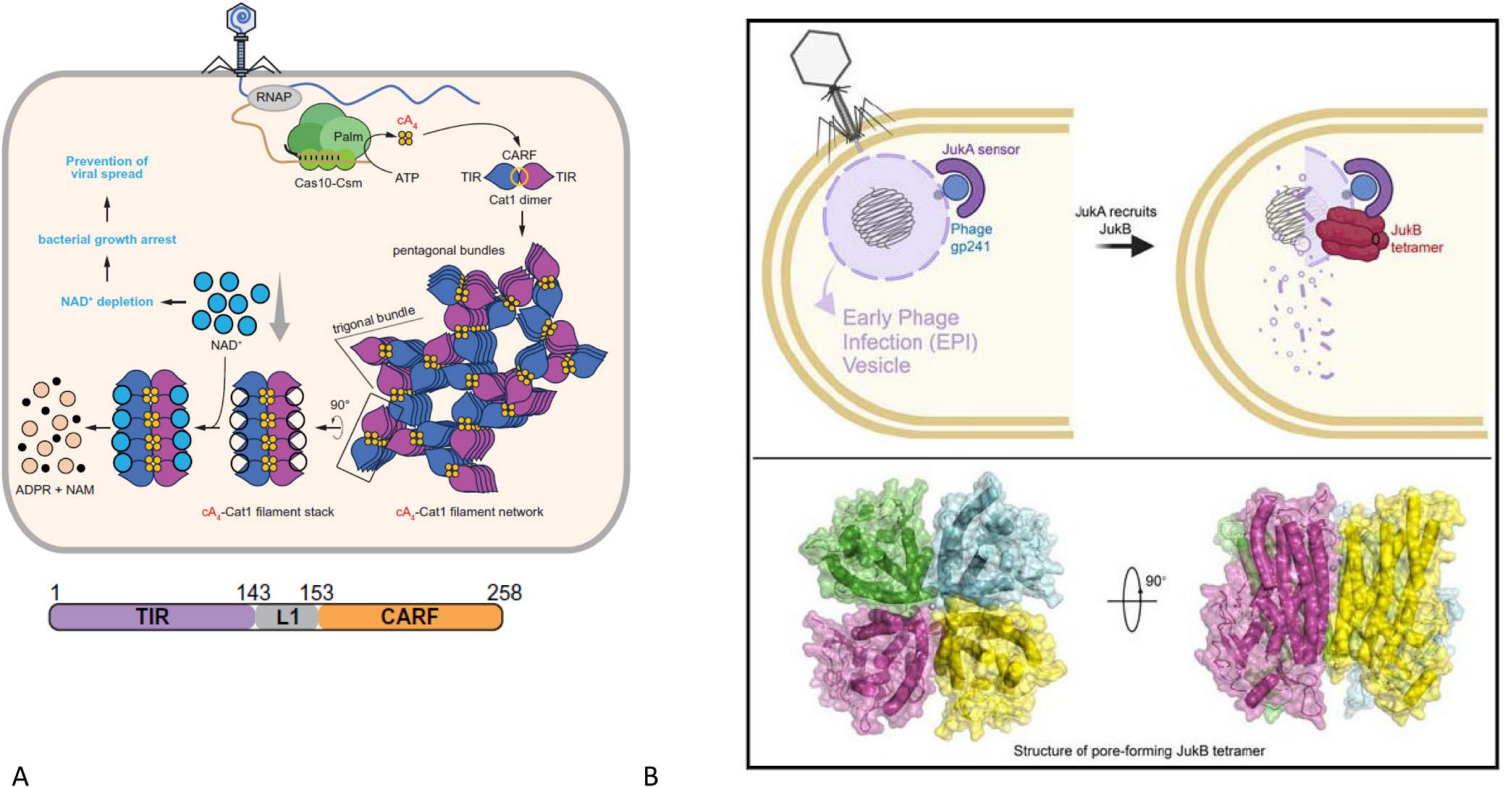
Model for Cat1 and jumbo phage killer APS action. Panel (A) Cat1 APS model. Upon detection of viral transcript by type III CRISPR‐Cas complex guided by CRISPR‐RNA, Cas10 synthesizes cyclic tetra‐adenylate (cA4) messenger molecules that bind to the Cat1 dimer to trigger dimer stacking and the formation of long filaments. These filaments further expand into a filament network formed by trigonal and pentagonal filament bundles. The stacked TIR domains of this filament assembly form the NAD^+^ binding pocket, which catalyses the cleavage of the substrate into ADPR and NAM. Depletion of cellular NAD^+^ causes bacterial growth arrest and prevents viral spread to the rest of the bacterial population. RNAP, RNA polymerase. Below is shown the domain architecture of Cat1. The protein contains an N‐terminal TIR domain followed by a linker (L1), and a C‐terminal CARF domain. Numbers indicate amino acids. Panel (B) The Juk APS. The Jumbo phage killer (Juk) system recognizes and targets phage protein gp241 in the early phage infection vesicle specific to phiKZ‐like jumbo phages with the sensor protein JukA. Sensor JukA bound to this early phage protein recruits JukB effector. JukB forms tetrameric pores that destabilize early phage infection vesicles, limiting early phage protein expression and aborts phage replication. The lower panel shows the structure of the pore‐forming JukB tetramer. Figure credit: Baca et al. (2025) and Yuping et al. (2025) under a Creative Commons Attribution License 4.0.

Type III CRISPR‐Cas 10 systems defend against phage infection by using an RNA‐guided complex that recognizes foreign transcripts and synthesizes cyclic oligoadenylate (cOA) messengers to activate CRISPR‐associated effectors (CARF). The effector function repertoire is large and comprises DNA or RNA cleavage, membrane depolarization, or ATP deamination. A new mechanism was investigated by Baca et al. (2025) when studying Cat 1, an effector containing a CARF domain fused to a Toll/interleukin‐1 receptor (TIR) domain (Figure 9A). Phage resistance was abolished when the targeting crRNA or the Cas 10 cyclase activity was lacking. The researchers demonstrated that the purified Cat1 catalysed in vitro the cleavage of NAD^+^ in the presence of the cA4 signal. Cat1 activity did not kill the cell; cell growth resumed 1 h later. In the absence of the cA4 signal, Cat1 is present as a dimer. In the presence of cA4, Cat1 forms long filaments with cA4 bound to the center serving as a molecular glue between adjacent Cat1 dimers. The filaments form networks of filaments consisting of trigonal and pentagonal bundles. The catalytic site for the cleavage of NAD^+^ was located in cryo‐EM studies to the lateral sides of the filaments. The catalytic site is formed by filamentation, and the enzymatic activity was enhanced by complex network formation. The CRISPR‐Cas 10 Cat1 system decreases intracellular NAD^+^ levels, leading to a bacterial growth arrest which decreased viral progeny production by 2 logs. The anti‐phage effect was greater when induced in the early phase of phage infection when phage replication still needs this essential cofactor. Destruction of cellular NAD^+^ is a common theme in bacterial APS, which will be illustrated with two examples.

## 21 Kongming: Using Noncanonical Inosine Nucleotides as Sensor to Activate NADase

Kongming APS in *E. coli* consists of three genes: KomA, a predicted adenosine deaminase; KomB, a HAM1‐like NTP pyrophosphatase and KomC, containing a SIR2‐like domain suggesting a NADase. Zeng et al. (2025) deciphered the mechanism of action for this APS. KomA deaminates dAMP to dIMP, a noncanonical inosine nucleotide. The phage‐encoded kinase enzyme DNK phosphorylates dIMP to dIDP and a cellular enzyme further phosphorylates it to dITP. HAM1‐like NTP pyrophosphatases are normally housekeeping cellular enzymes to remove noncanonical nucleotides. However, KomB has lost this phosphatase activity and serves in this APS as a phage sensor detecting dITP which needs the phage DNK for synthesis. KomB forms a complex with KomC. Binding of dITP to KomB leads to a conformational change that activates the enzymatic activity in KomC which cleaves NAD^+^ depriving the cell of this essential cofactor for survival. Phage T5 encodes DNK but is not susceptible to Kongming inhibition. T5 encodes an enzyme Dmp which dephosphorylates dAMP to dA suppressing the synthesis of the phage sensor dITP demonstrating measures and countermeasures in the genetic arms race between bacteria and phages.

## 22 Thoeris: TIR‐Domain Proteins Elaborating Distinct Chemical Signals

The prototype Thoeris APS was described by Ofir et al. (2021). In *Bacillus cereus* it consists of two genes, *thsA* encoding two domains, SIR2 and SLOG, followed by one or more thsB genes that encode a TIR domain known as Toll/interleukin‐1 receptor, known from plant cells inducing cell suicide by a hypersensitive response. ThsB is a phage sensor that produces 45 min after phage infection an isomer of a cyclic adenine diphosphate ribose (cADPR). Different ThsB recognize different *Bacillus subtilis* phages. The SLOG domain of ThsA senses the signal which drives oligomerization of ThsA. Oligomerization activates the NADase, encoded by the SIR2 domain, cleaving NAD^+^, killing the cell and aborting the phage infection. Recently, further types of Thoeris APS were reported. Type IV in *Pseudomonas* (Rousset et al. 2025) consists of a two gene operon encoding a TIR domain protein and a caspase‐like protease. When expressed in *E. coli* it protects against phage T6. At low MOI, the cell survives the infection; at high MOI the cell dies but does not release progeny phage; amino acid substitutions in either of the two proteins abolish phage resistance. However, no decrease in NAD^+^ level was observed. The researchers observed that the caspase‐like protease cleaves elongation factor Tu (EF‐Tu) after activation by a small metabolite produced by TIR. The caspase‐like protein is a promiscuous arginine‐specific protease that cleaves multiple proteins. None of the signalling molecules from type I to III Thoeris systems activated the caspase from type IV Thoeris. By a combination of chromatography and a peptide cleavage assays in a fluorescent reporter system, the researchers could isolate the activator compound. Chemical analysis identified a so far unknown natural product (Figure 1C). Structural modelling demonstrated that the caspase‐like protein forms a dimer forming a pocket into which the N7‐cADPR signal fits. Mutations of side chains involved in ligand binding also abolished phage resistance. Once infection is sensed (the mechanism remains to be defined), the TIR‐domain protein utilizes NAD^+^ as a substrate to generate the cyclized ADPR signalling molecule.

Researchers analysed Thoeris II system (Sabonis et al. 2025). Chemical analysis of cell lysates, together with a phage sponge protein adsorbing the signalling molecule (and thereby inactivating the anti‐phage function of Thoeris II) helped to identify its chemical nature. In contrast to diverse nucleotide signals identified in Type I Thoeris system, Thoeris II systems use histidine conjugated to ADP‐ribose as its signalling molecule (His‐ADPR) (Figure 1D). This signal binds to the MACRO domain of ThsA, inducing its oligomerization. The effector contains transmembrane domains and its oligomerization destabilizes the inner cell membrane, leading to cell death and thereby aborts phage amplification.

## 23 Integrons: A Low Cost Biobank of APS

Mobile integrons (MI) are genetic elements that have been mobilized by transposons onto plasmids and play important roles in bacterial adaptation to changing environmental conditions. Integrons consist of an integrase gene and recombination site in the integron (*attI*) for integration. Integrons can capture, store, shuffle and express promoterless gene cassettes terminated by recombination sites (*attC*). MI are best known for carrying almost 200 resistance genes against most antibiotic families. However, MIs also harbour gene cassettes of unknown function. Researchers from Madrid cloned these cassettes in *E. coli* which they challenged with various coliphages. At least a third of the cloned cassettes encoded phage resistance systems; most of them are new and contain proteins with unknown domains (Kieffer et al. 2025). Mobile integrons can reach new hosts through conjugation. When transferred to *Klebsiella pneumoniae* or *Pseudomonas aeruginosa* , the cloned cassettes conferred resistance to several phages, demonstrating their activity in different species. Some systems inhibited prophage activation; however, none showed anti‐plasmid activity. Integrons are low‐cost platforms that can modulate the expression of genes in the array by shuffling positions with respect to a promotor. By this mechanism, MI can modulate the fitness cost to the bacterial cell, providing phage resistance on demand. The researchers expressed the concern that MI cannot only compromise the use of antibiotics but could also interfere with phage therapy approaches because MI can accumulate resistance to both phages and antibiotics.

Sedentary chromosomal integrons (SCIs) in contrast are not mobile and encode large arrays of gene cassettes of unknown function. A bioinformatic analysis of 30,000 bacterial genomes revealed 783 antiphage systems within integron cassettes. Researchers from the Pasteur Institute in Paris cloned 88 SCI cassettes of unknown function from the seventh pandemic *Vibrio cholerae* strain (Darracq et al. 2025). When tested against Vibriophages and coliphages, 16 cassettes encoded antiphage systems. Eleven resembled known APS while 5 were novel. Antiphage proteins from SCI tended to be small, down to 63 aa length. When relocating an antiphage function from a more distal to the first integron position next to the integron promoter, transcription of that gene and phage protection was activated. The researchers developed a model according to which SCI represents a biobank of low‐cost non‐expressed anti‐phage functions that can be activated by recombination processes under phage selection.

## 24 Killing Jumbo Phages

*Pseudomonas* phage phiKZ is a jumbo phage that assembles a membrane bound early phage infection (EPI) vesicle where early phage transcription takes place. Subsequently, phiKZ builds a proteinaceous nucleus‐like structure where phage DNA replication occurs protected from cellular DNases. Researchers described a jumbo phage killer (Juk) immune system consisting of a broadly conserved sensor (JukA) that recognizes phage phiKZ early protein gp241 (Yuping et al. 2025) (Figure 9B). JukA sensor proteins are found in 21% of bacterial genomes from proteobacteria and cyanobacteria while JukB effector proteins are only found in 1% of these genomes suggesting that JukA can combine with different effectors. Juk immune proteins act rapidly to block essential early events of the phage life cycle occurring at the EPI vesicle, leading to phage DNA degradation. Upon phage infection, JukA and JukB rapidly clustered at cell poles. Phage gp241 binds JukA and enhances JukB association. Crystallography of JukB revealed a pore‐forming tetramer. The complex of JukAB/gp241 permeabilized liposomes. The complex also permeabilizes the EPI vesicle membrane but not the inner bacterial cell membrane; therefore, the infected cell survives phage infection. The EPI vesicle permeabilization leads to phage DNA degradation probably by bacterial nucleases. Bioinformatic analysis showed that JukA is associated in other bacteria with restriction‐modification enzymes or nucleases suggesting the combination with different effectors.

## 25 Gasdermin: A Precursor of a Mammalian Innate Immune System Found in Bacteria

Gasdermin proteins form large membrane pores in human cells that induce lytic cell death. Gasdermin pore formation is triggered by caspase‐mediated cleavage during inflammasome signalling in the defence against pathogens. Gasdermin homologues and their activators were also detected in bacteria where they defend against phages, pointing to an ancient origin of this pathogen defence system (Johnson et al. 2022). Wein et al. (2025) analysed the gasdermin system from *Lysobacter*. The operon consists of four genes: gasdermin, followed by two trypsin‐like proteases and an NLR‐like gene (nucleotide binding‐domain, leucine‐rich repeat‐containing proteins). The second protease contains an N‐terminal CARD (Caspase recruitment domains). The researchers cloned and expressed the gasdermin operon in *E. coli* . Following phage T6 infection, the bacterial gasdermin protein is activated by proteolytic cleavage and oligomerizes into pores that cause cell death, which prevents phages from completing their replication cycle. Deletion of CARD in the second protease gene abolished cleavage of gasdermin and phage defence. Also, the ATPase domain in the second protease is essential for phage defence. The CARD domain, in addition, mediates protein–protein interaction with the NLR‐like protein. The proteases associated with bacterial gasdermins were shown in vitro to cleave the bacterial gasdermin. Further insight came from phage escape mutants. Phage T6 escapers showed a mutation in the *rIIB* phage gene, suggesting this phage protein as a trigger of this APS. Indeed, coexpression of rIIB with the gasdermin system in the absence of phage also caused gasdermin cleavage, aggregation and membrane permeabilization. Deletion of CARD from the protease again suppressed all three activities. Apparently, detection of the rIIB protein activates the gasdermin defence system. The *rIIB* gene is found in 5% of phages from the database, where it is encoded in an operon with the *rIIA* gene. The *rIIAB* operon allows T‐even phages to overcome the APS *RexAB* from *E. coli* . The researchers concluded that their observations are a testimony of an arms race between bacteria and phages, where phages encoding a protein that serves to evade one immune system become sensitive to other defence systems by this very protein.

## 26 Common Themes Shared Between Bacterial and Mammalian Antiviral Systems

**FIGURE 10:**
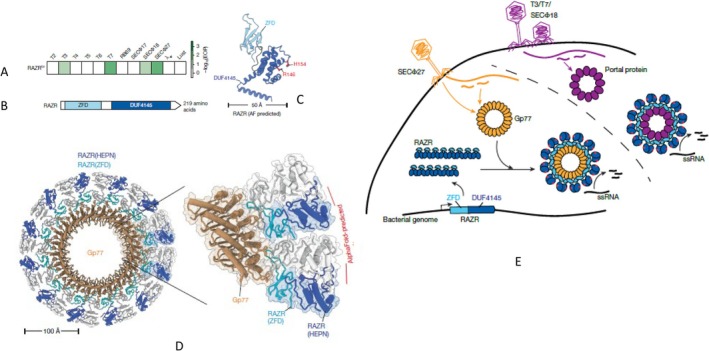
Model for the ring‐activated zinc‐finger RNase (RAZR) APS action. Panel (A) Efficiency of plating for selected phages indicated at the top on cells containing the RAZR APS, expressed as negative logarithm according to the colour scale at the right. Panel (B) Domain topology of RAZR; ZFD, zinc‐finger domain; DUF4145 domain resembling HEPN RNase. Panel (C) Predicted structure for the RAZR monomer, the domains and the active site residues are indicated. Panel (D) The 24‐meric phage Gp77 ring in contact with 24 RAZR subunits; the positions of the ZFD and HEPN domains are indicated by different blue shading (left), and a detailed view of 4 protomers of Gp77 and 4 protomers of RAZR, coloured by domains is shown at the right. The double ring structure contains an inner ring composed of phage Gp77 subunits and outer ring of RAZR subunits. Panel (E) Model for RAZR action. RAZR is transcribed from the bacterial genome and translated into linear oligomers. Phage phi27 forms a Gp77 ring structure or phages T3, T7 and SECphi18 form rings with their portal proteins. RAZR aggregates on these phage ring protein complexes forming a second outer ring structure which activates the RNase function of DUF4145 degrading ssRNA, thus blocking protein translation and aborting phage infection. Figure credit: Zhang, Lyu, et al. (2026) under a Creative Commons Attribution License 4.0.

An exciting feature of bacterial APS is their resemblance with innate antiviral systems in eukaryotes including mammalian systems as demonstrated by gasdermin. Additional examples can be cited. The formation of large, high‐order macromolecular complexes is a common theme in eukaryotic immunity: for example, during viral infection RNase L dimerizes, leading to activation of its HEPN domains (higher eukaryote and prokaryotes nucleotide‐binding RNase) and cleavage of viral and endogenous RNAs. The restriction factor TRIM5a can form higher‐order oligomers, templated by the geometry of retroviral cores, to block viral replication. Very similar mechanisms have recently also been described in APS (Zhang, Lyu, et al. 2026) (Figure 10). The researchers characterized ring‐activated zinc‐finger RNase (RAZR) from *E. coli* that restricted several coliphages. The APS consisted of a single protein containing two domains: an N‐terminal zinc finger domain (ZFD) and a C‐terminal HEPN domain. Overexpressed RAZR is not toxic to *E. coli* but in the presence of phage SECφ27 infection, cell death and abortive infection was observed. The researchers determined the phage protein that triggers the activation of RAZR: for SECφ27 it was Gp77 and for phage T3 and T7 it was the phage portal protein. The two phage protein groups do not share sequence homology. However, both have roles in phage genome packaging and form a ring structure consisting of 24 copies of Gp77 or 12 copies of the portal protein. In cryo‐EM analysis, RAZR on its own forms a linear oligomer. In the presence of the Gp77 ring structure, RAZR adds a further ring layer surrounding the inner phage ring. The ZDF domain of RAZR mediates the contact with the phage ring and the HEPN domain constitutes the outer rim of this three‐layered ring structure. Phage escape mutants showed aa substitutions in interacting regions between the phage ring and ZFD. The researchers deduced that ZFD of RAZR serves as an infection sensor that transmits the phage protein binding information to the HEPN domain, activating the RNase. Each individual HEPN domain contains only part of the active site of the RNase and oligomerization of HEPN is needed for enzymatic activity. RAZR blocks protein synthesis by cleaving non‐specifically tRNA, rRNA and mRNA. Specificity is conferred to the system by the fact that the bacterial cell contains only a single ring protein structure, the flagellar basal body, which measures 260 Å and not 170 Å as the phage ring structure. RAZR homologues from different bacteria vary in their ZFD domain which thus assures phage specificity of this APS.

**FIGURE 11:**
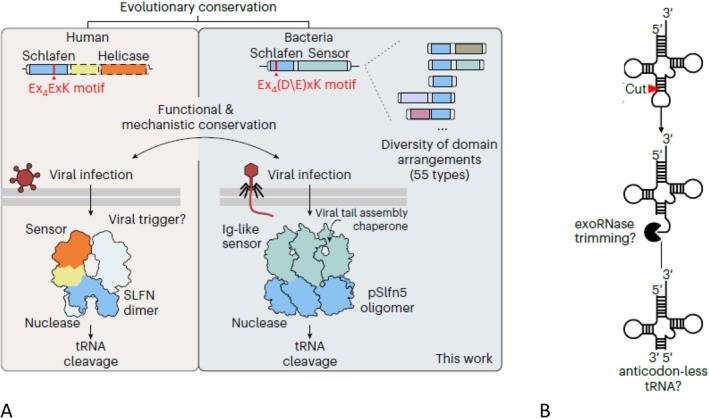
Evolutionary conservation of the human anti‐viral protein Schlafen and the bacterial anti‐phage Schlafen‐like proteins. Panel (A) Left: Structure prediction for the human SLFN11 dimer with the nuclease N‐terminal Schlafen domain (blue), the linker (yellow) and the C‐terminal helicase sensor domain (brown). Viral infection triggers the Schlafen complex and activates tRNA cleavage by the nuclease domain. Right, at top: domain topology of the bacterial Schlafen‐like protein with the Schlafen nuclease domain and its active site residues and the Ig‐like sensor region triggered by a phage tail chaperone. This leads to nuclease oligomerization activating tRNA cleavage. The nuclease domain can be fused to a diverse array of phage‐sensing domains, indicating by different colours at the top right. Panel (B) Proposed mechanism of the tRNA cleavage. Schlafen‐like nuclease cuts the tRNA, followed by exoRNase trimming which leads to an anticodon‐less tRNA. Figure credit: Perez Taboada et al. (2026) under a Creative Commons Attribution License 4.0.

Another case of a striking parallel between mammalian and bacterial antiviral responses is provided by the human Schlafen protein family (Slfn), which acts as interferon‐inducible antiviral factors, cleaving tRNA and rRNA. Researchers identified thousands of Slfn homologues with a conserved active site in bacterial genomes, many located in defence islands (Perez Taboada et al. 2026) (Figure 11). The Slfn homologue was mostly fused to another protein domain, which was variable and conferred the phage specificity of the system. The researchers demonstrated anti‐phage T5 activity for one of the bacterial Schlafen proteins and identified the phage T5 tail assembly chaperone as viral trigger. In their investigated system, a C‐terminal immunoglobulin‐like domain of Slfn acted as phage sensor. After association with the viral trigger, an oligomerization of the Schlafen protein was induced. The N‐terminal part of the bacterial Schlafen protein is a phage‐activated tRNase, cleaving in the anticodon loop followed by further trimming of the tRNA fragments.

Further evidence for ancestral immunity functions found in both prokaryotic and eukaryotic systems was provided by the analysis of the Thoeris 2 APS in *Bacillus cereus* (Tamulaitiene et al. 2024). The TIR‐domain protein ThsB senses phage infection and becomes activated and synthesizes an isomer of cyclic ADP ribose (Figure 1E). This signalling molecule binds to the SLOG domain (a fold binding nucleotides or signalling molecules) of the effector protein ThsA, which leads to the assembly of a micrometre‐long ThsA helical filament. The filament formation activates the SIR2 (silent information regulator 2) NADase that depletes NAD^+^ killing the cell and thus aborts phage infection. The researchers noted that the budding yeast sirtuin protein Sir2, an NAD^+^‐dependent histone deacetylase, displays a SIR2 domain activation and regulation strategy shared between prokaryotes and eukaryotes. In a reverse process to that of Perez Taboada et al. (2026) looking for bacterial homologues of human proteins, researchers at the Pasteur Institute screened for eukaryotic homologues of bacterial SIR2 anti‐phage proteins (Bonhomme et al. 2025). They identified such proteins across the tree of life involved both in housekeeping functions (sirtuin) and in immune function (SIRim). One form of SIRim was found in vertebrates including humans. SIRal mediates the up‐regulation of interferon‐stimulated genes (ISGs) and of proinflammatory cytokines and restricts herpesvirus and *Salmonella* infections in macrophages. The researchers concluded that the conservation of immune modules across the entire tree of life points to ancestral immunity functions.

In the present context, one might also quote APS using ubiquitin‐like proteins. Ubiquitination of proteins is not only central to the protein degradation pathway in humans relying on a cascade of enzymatic reactions by E1, E2 and E3 proteins, but also important in pathways of innate immunity. The interferon‐stimulated gene 15 (ISG15) is a ubiquitin‐like (Ubl) protein with two fused Ubl domains protecting against influenza A virus and HIV‐1. Hör et al. (2024) described a bacterial BilABCD operon where Bil stands for bacterial ISG15‐like system. BilA encodes a Ubl where each of the fused two Ubl domains overlap the human ubiquitin tertiary structure. BilB and BilD encode functional analogs of E2 and E1 proteins, respectively. BilC corresponds to a deubiquitinase that is also essential for phage resistance. Cells containing the BilABCD operon neither survive phage infection nor lyse prematurely to terminate phage production. However, the released phages either lacked a tail or those containing a tail showed Ubl‐addition to the phage central tail fibre which obstructs the receptor‐binding domain, leading to failed phage adsorption. Chambers et al. (2024) describe a bacterial E1–E2–Ubl complex that reveals striking architectural parallels with the eukaryotic ubiquitination machinery, and they suggested that this pathway first arose in bacteria.

## 27 Outlook

The molecular underpinning of bacterial phage defence systems is currently a very active research area providing a flood of data that illustrate an unanticipated diversity of prokaryotic anti‐viral systems. New reports continue to add fascinating facets of how phage infections are detected and bacterial counter‐measures are mounted. One gets the impression that only the tip of a much larger iceberg of defence systems has been characterized. Much remains to be explored which will reveal intriguing new molecular mechanisms developed by prokaryotes to deal with the challenge of phage predation. The underlying interesting molecular biology will certainly lead to innovative new biotechnological tools. One can already see how the exchange of modular elements of defence subfunctions can create an incredible array of anti‐viral strategies. However, as frequently in biology, behind the dizzying variety of solutions, one starts to decipher common themes of the anti‐viral response shared between prokaryotic and eukaryotic organisms, up to innate immunity mechanisms functional in vertebrates and even humans. One important aspect is however lacking in the present review: the counter‐attack of phages against bacterial defence systems. The arms race between viruses and cells has shaped important aspects of organismic evolution, and the next review will explore phage counter‐attacks as documented in recent publications.

## Author Contributions

**Harald Brüssow:** conceptualization, investigation, writing – original draft.

## Funding

The author has nothing to report.

## Conflicts of Interest

The author declares no conflicts of interest.

## Data Availability

Data sharing not applicable to this article as no datasets were generated or analysed during the current study.
